# Structural and functional alterations in postmenopausal women with insomnia: an MRI study of Eight-Section Vajra Exercise intervention effects

**DOI:** 10.3389/fnins.2025.1622756

**Published:** 2026-01-30

**Authors:** Xiangbin Chen, Kun Wang, Xiaoying Xu, Yuxi Li, Yu Gao, Jiamin Yuan, Biyun Xu, Shijun Qiu, Fei Tan, Zhimin Yang

**Affiliations:** 1 The Second Clinical College of Guangzhou University of Chinese Medicine, Guangzhou, China; 2 The First Clinical Medical College, Guangzhou University of Chinese Medicine, Guangzhou, China; 3 Guangzhou Yuexiu District Renmin Street Community Health Service Center, Guangzhou, China; 4 The Second Affiliated Hospital of Guangzhou University of Chinese Medicine, Guangzhou, China; 5 Department of Radiology, The First Affiliated Hospital of Guangzhou University of Chinese Medicine, Guangzhou, China; 6 State Key Laboratory of Traditional Chinese Medicine Syndrome, Guangzhou, China; 7 Guangdong Provincial Hospital of Chinese Medicine, Guangzhou, China

**Keywords:** postmenopausal, women, insomnia, MRI, Eight-Section Vajra Exercise, neuroimaging

## Abstract

**Background:**

Postmenopausal women exhibit heightened vulnerability to chronic insomnia due to estrogen decline and age-related neural alterations. While non-pharmacological interventions are preferred for long-term management, the neuroplastic mechanisms underlying exercise-based therapies remain poorly characterized.

**Methods:**

This study examines the effects of Eight-Section Vajra Exercise (ESVE) on brain structure and function in postmenopausal women with insomnia (PMWI) using multimodal MRI. A 12-week ESVE training program was completed by PMWI patients, followed by clinical assessments (PSQI, ISI, PHQ-9, GAD-7, FSS, MoCA) and neuroimaging (fMRI and structural MRI). Data analysis involved gray matter volume (GMV), ALFF/fALFF, ReHo, degree centrality (DC), and functional connectivity (FC) using advanced MRI processing techniques (CAT12, SPM12, DPABI). Group comparisons and correlations were adjusted for age, education, and intracranial volume.

**Result:**

Among the 24 PMWI patients and 30 healthy controls (HCs), baseline measures showed significantly worse sleep and mood scores in PMWI. Resting-state fMRI revealed reduced ALFF/fALFF in the right precentral gyrus and decreased ReHo in sensorimotor areas. Changes in functional connectivity (FC) were noted, with altered connections between precentral gyrus, temporal and parietal regions. After 12 weeks of ESVE, 78.95% of PMWI patients were medication-free, with post-treatment fMRI showing improved neural activity and connectivity, correlating with clinical improvement. Exercise adherence positively correlated with sleep quality improvement (*r* = 0.508–0.594, *P* < 0.05). Responders showed significant improvements in sleep compared to non-responders (*P* < 0.05).

**Conclusion:**

ESVE alleviates postmenopausal insomnia through PreCG-centered sensorimotor-visual network reorganization, potentially compensating for estrogen-dependent neurocircuitry vulnerabilities. Exercise-induced GMV increases in occipitotemporal regions suggest enhanced sleep-related memory consolidation. Our findings indicate that ESVE is a potential neuromodulatory intervention and identify PreCG-MOG connectivity as a promising biomarker for personalized insomnia management.

## 1 Introduction

Insomnia affects approximately 10–30% of the global adult population (Morin et al., 2015), with postmenopausal women accounting for nearly half of this proportion (43–48%) (Kravitz et al., 2003), which is particularly related to postmenopausal women being vulnerable to hormonal fluctuations and sleep structure changes associated with aging (Jehan et al., 2015). Postmenopausal women experience hormonal changes due to ovarian function decline, leading to menstrual cessation and vasomotor symptoms, which may reduce quality of life and work efficiency (Hartz et al., 2013; Woods and Mitchell, 2011). This population commonly experiences hot flashes, night sweats, sleep disorders, mood disorders, and cognitive decline, with vasomotor symptoms and mood disturbances leading to frequent nocturnal awakenings, difficulty in sleep restoration, and rumination, thus perpetuating the insomnia cycle (Baker et al., 2018). These sleep disturbances not only impair cognitive function and emotional regulation but also significantly increase the risk of cardiovascular disease, metabolic disorders, and neurodegenerative conditions (Van Der Zweerde et al., 2019; Wardle-Pinkston et al., 2019; Miller and Howarth, 2023).

Current therapeutic approaches include both pharmacological and non-pharmacological interventions. While hormone therapy is commonly prescribed for menopausal symptoms, studies have revealed increased risks of cardiovascular disease, breast cancer, gallstones, and dementia among treated women (Flores et al., 2021; Rossouw et al., 2002). The mounting evidence of adverse health risks associated with menopausal hormone therapy has led patients and clinicians to question its long-term safety and seek alternative treatment strategies (Mehta et al., 2021).

Therefore, many women are seeking relief from menopause-related symptoms through complementary and alternative medicine to avoid these adverse effects. Among various non-pharmacological interventions, exercise therapy has emerged as a promising option due to its safety profile and effectiveness. Exercise intervention not only offers potential health benefits comparable to pharmacological treatments, but also has no significant adverse effects typically associated with long-term medication use. Recent meta-analyses have demonstrated that programmed exercise interventions can effectively improve sleep quality in postmenopausal women (Rubio-Arias et al., 2017). However, controversy exists regarding yoga practice, as systematic reviews found no significant effects of yoga intervention on either sleep quality or insomnia severity in postmenopausal women (Rubio-Arias et al., 2017; Wang et al., 2020). The Eight-Section Vajra Exercise (ESVE), originating from Taoist monastic practice (Mi, 2013.), is a mind-body exercise that integrates physical movements with meditative elements, and its simple and accessible nature makes it particularly suitable for postmenopausal women, suggesting its potential as an alternative exercise intervention for sleep improvement in this population.

Advances in Magnetic Resonance Imaging (MRI) have enabled non-invasive and sensitive measurement of pathological changes in the cortical and subcortical brain parenchyma. In recent years, the imaging research about the brain of postmenopausal women has gradually been paid attention by researchers. Studies have shown that brain volume changes on imaging with increasing age, and postmenopause shows changes in specific brain structures due to hormonal and estrogen changes (Mosconi et al., 2021), especially in the frontal cortex, followed by the hippocampus and temporal cortex. Structural magnetic resonance imaging studies have reported reduced gray matter volume in assisted motor areas, inferior frontal gyrus, olfactory cortex, and superior temporal gyrus in postmenopausal women compared with premenopausal women (Zhang et al., 2018), while resting-state functional magnetic resonance imaging (fMRI) studies have revealed altered functional connectivity patterns in these populations. A similar study demonstrated accelerated hippocampal volume reduction in postmenopausal women (Goto et al., 2011). These regions are known to play a central role in a variety of behavioral and cognitive functions, being associated with depressive states, poor sleep quality, and decreased executive function (Armstrong et al., 2020; Sigurdsson and Duvarci, 2015; Rubin et al., 2014) We combined Voxel-Based Morphometry (VBM) for structural analysis of Gray Matter Volume (GMV) (Ashburner and Friston, 2000) and resting-state functional MRI (rs-fMRI) for examining intrinsic neural activity. While voxel-based morphometry (VBM) provides valuable insights into gray matter volume changes, advanced diffusion-weighted imaging (DWI) analysis tools could offer complementary information about microstructural alterations at the white matter level, which may be considered for future investigations. To comprehensively characterize brain function, we utilize complementary measures including Amplitude of Low-Frequency Fluctuations (ALFF), fractional ALFF (fALFF), Regional Homogeneity (ReHo), degree centrality (DC) and whole-brain seed-based functional connectivity (FC) (Zang et al., 2007; Zou et al., 2008; Jiang and Zuo, 2016; Buckner et al., 2009). These indicators provide multi-dimensional assessment of brain characteristics, enabling sensitive detection of regional abnormalities and enhanced understanding of structure-function relationships in both healthy and pathological states to give a better understanding of the functional changes in the brain of postmenopausal women.

This study explores how insomnia in postmenopausal women, influenced by hormonal and neural changes, might be helped by ESVE. It aims to understand baseline brain differences and the potential benefits of ESVE on both sleep quality and brain function using advanced brain imaging techniques. Given the limited understanding of brain network alterations in PMWI, we hypothesize that: (1) PMWI patients will exhibit reduced gray matter volume in frontal and temporal regions compared to healthy controls; (2) PMWI patients will show decreased ALFF, fALFF, and ReHo values in sensorimotor and cognitive control regions; (3) PMWI patients will demonstrate altered functional connectivity patterns, particularly in networks involved in sleep regulation and motor control; and (4) A 12-week ESVE intervention will normalize these brain alterations, with improvements in sleep quality correlating positively with restored brain connectivity patterns, particularly in precentral gyrus-centered networks. Our study uses multimodal magnetic resonance imaging (MRI) to test these hypotheses, employing a range of metrics to map the neural patterns of insomnia, evaluate the intervention’s efficacy, and explore the link between brain changes and therapeutic responses, ultimately aiming to develop personalized treatment strategies based on neuroimaging biomarkers.

## 2 Materials and methods

### 2.1 Participants

We enrolled PMWI patients from two centers (First Affiliated Hospital of Guangzhou University of Chinese Medicine and Guangdong Hospital of Traditional Chinese Medicine) between September 2023 and June 2024, PMWI recruited from the local community. Eligible participants were postmenopausal women aged 40–70 years with a diagnosis of insomnia, Pittsburgh Sleep Quality Index (PSQI) scores > 7, and capacity to complete all assessments. Healthy controls (HCs) were required to be free of insomnia disorders. PMWI patients and healthy controls were matched for age (± 5 years) and education level (± 2 years).

We excluded candidates who: (i) were unable to complete three 30-min ESVE sessions; (ii) had secondary insomnia from underlying conditions (malignancy, autoimmune disorders), medications (corticosteroids, chemotherapy), or chronic pain; (iii) presented with severe sleep apnea, restless legs syndrome, or parasomnia history (personal/familial); (iv) had intellectual disability, psychiatric disorders, substance use disorders, or suicidal tendencies; (v) showed unstable vital signs; (vi) required co-sleeping with children; (vii) exhibited severe anxiety (GAD-7 ≥ 15) or moderate-to-severe depression (PHQ-9 ≥ 15); (viii) participated in recent clinical trials; (ix) had prior ESVE experience; (x) demonstrated severe insomnia (ISI ≥ 22); (xi) used medications including melatonin receptor agonists (agomelatine, ramelteon, tasimelteon), atypical antipsychotic medications (olanzapine, quetiapine), other antihistamines (diphenhydramine, chlorpheniramine, promazine, etc.), melatonin, and valerian; or (xii) had MRI-incompatible implants. Sleep disorders were systematically excluded through clinical interviews and medical history review, though the absence of polysomnography represents a study limitation.

Assessment instruments included the Pittsburgh Sleep Quality Index [PSQI; measuring seven components: subjective sleep quality (SSQ), sleep latency (SL), sleep duration (SDu), sleep efficiency (SE), sleep disturbances (SD), use of sleep medications (SM), and daytime dysfunction (DD)] and Insomnia Severity Index (ISI) for sleep evaluation. We used the Patient Health Questionnaire-9 (PHQ-9) and Generalized Anxiety Disorder-7 (GAD-7) to assess emotional status, the Fatigue Severity Scale (FSS) for fatigue assessment, and the Montreal Cognitive Assessment (MoCA) for cognitive function.

**FIGURE 1:**
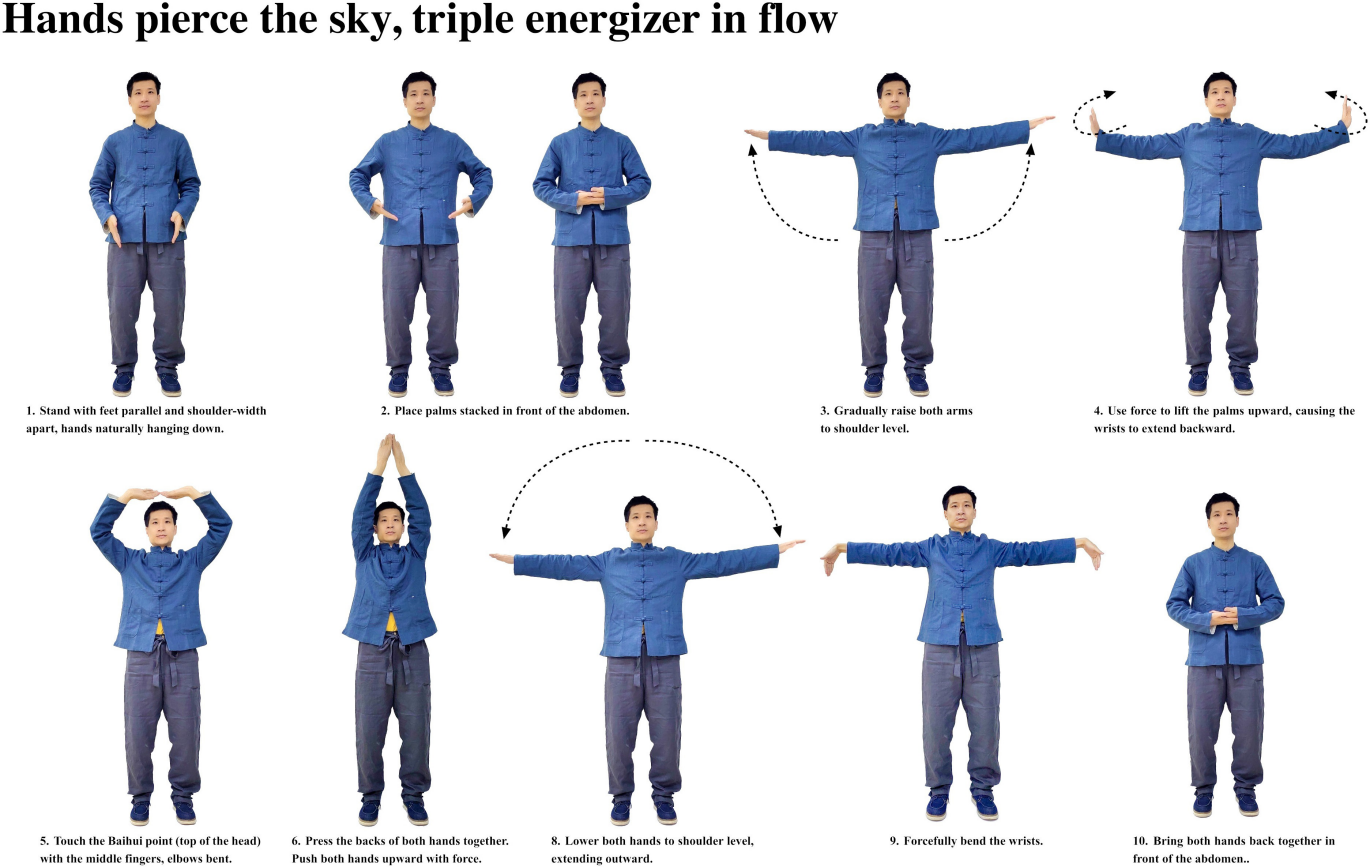
Hands pierce the sky, triple energizer in flow.

**FIGURE 2:**
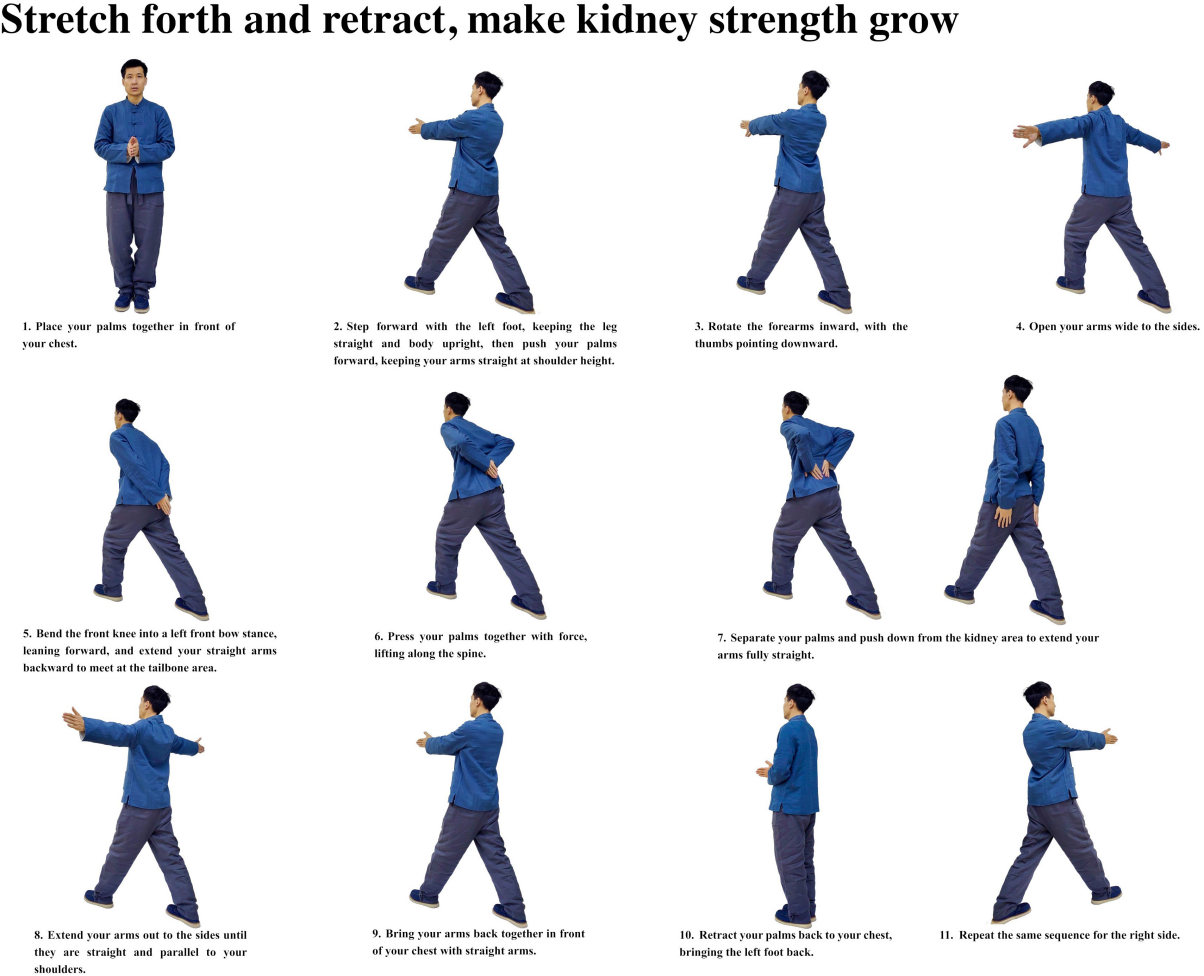
Stretch forth and retract, make kidney strength grow.

**FIGURE 3:**
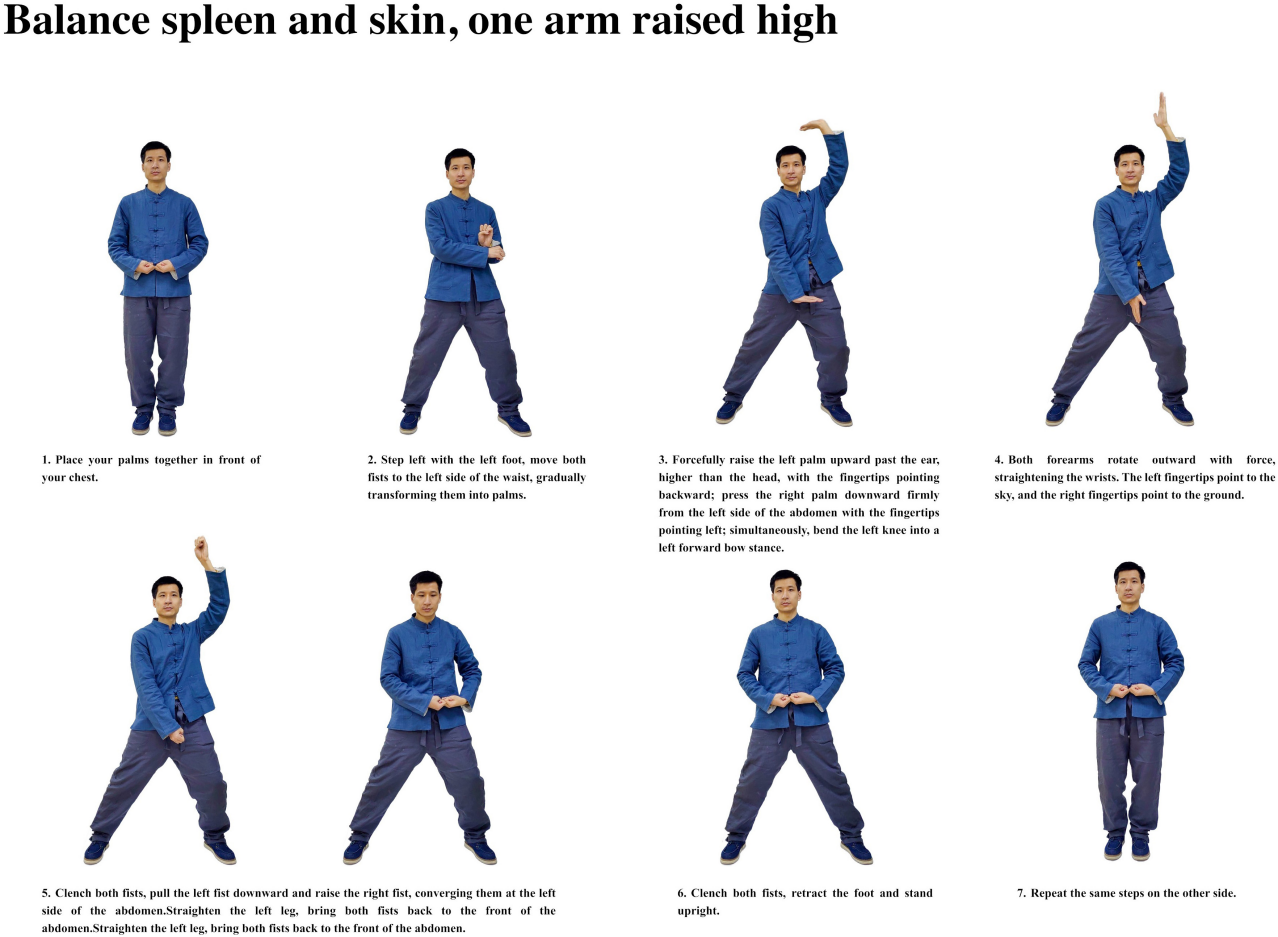
Balance spleen and skin, one arm raised high.

**FIGURE 4:**
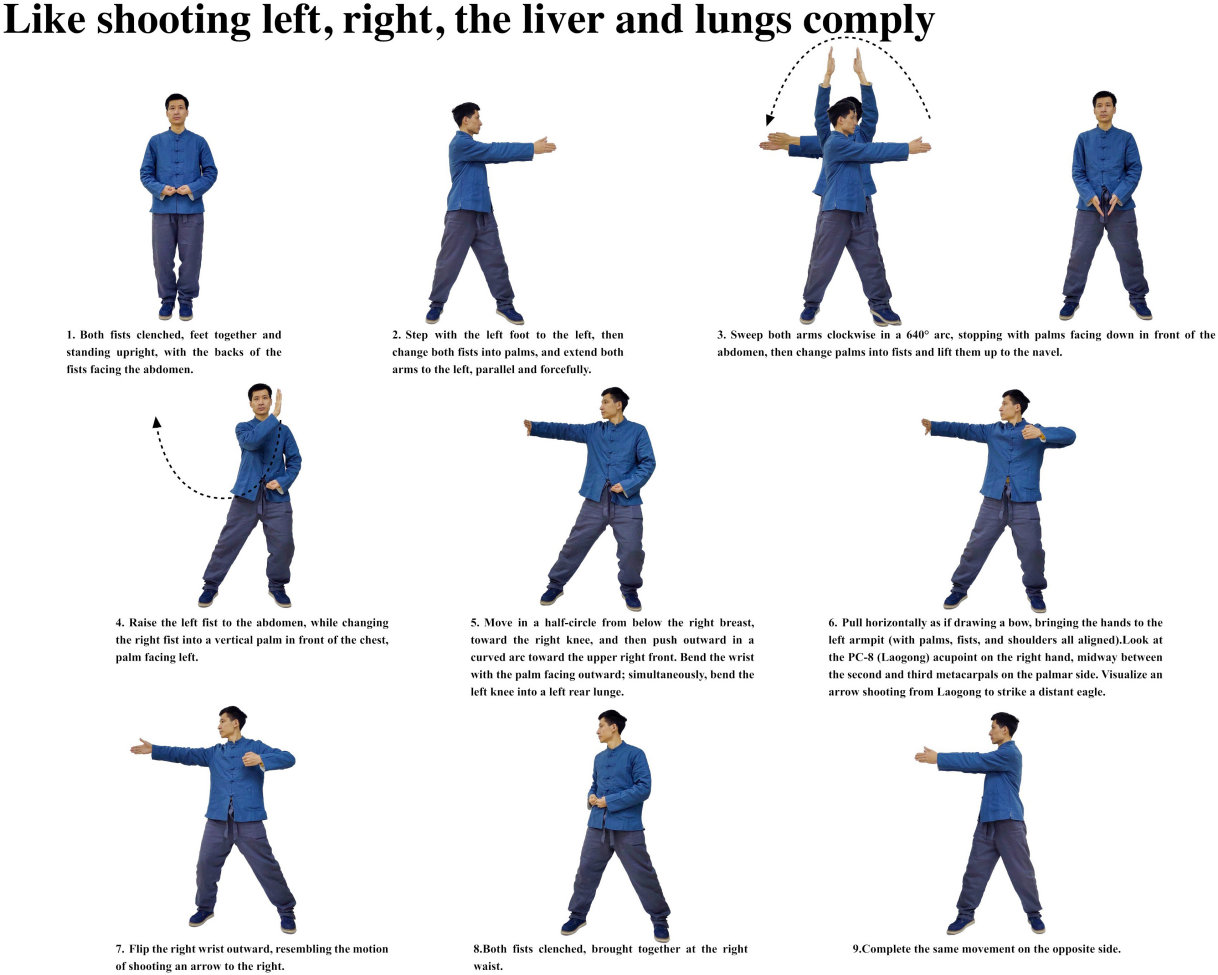
Like shooting left, right, the liver and lungs comply.

**FIGURE 5:**
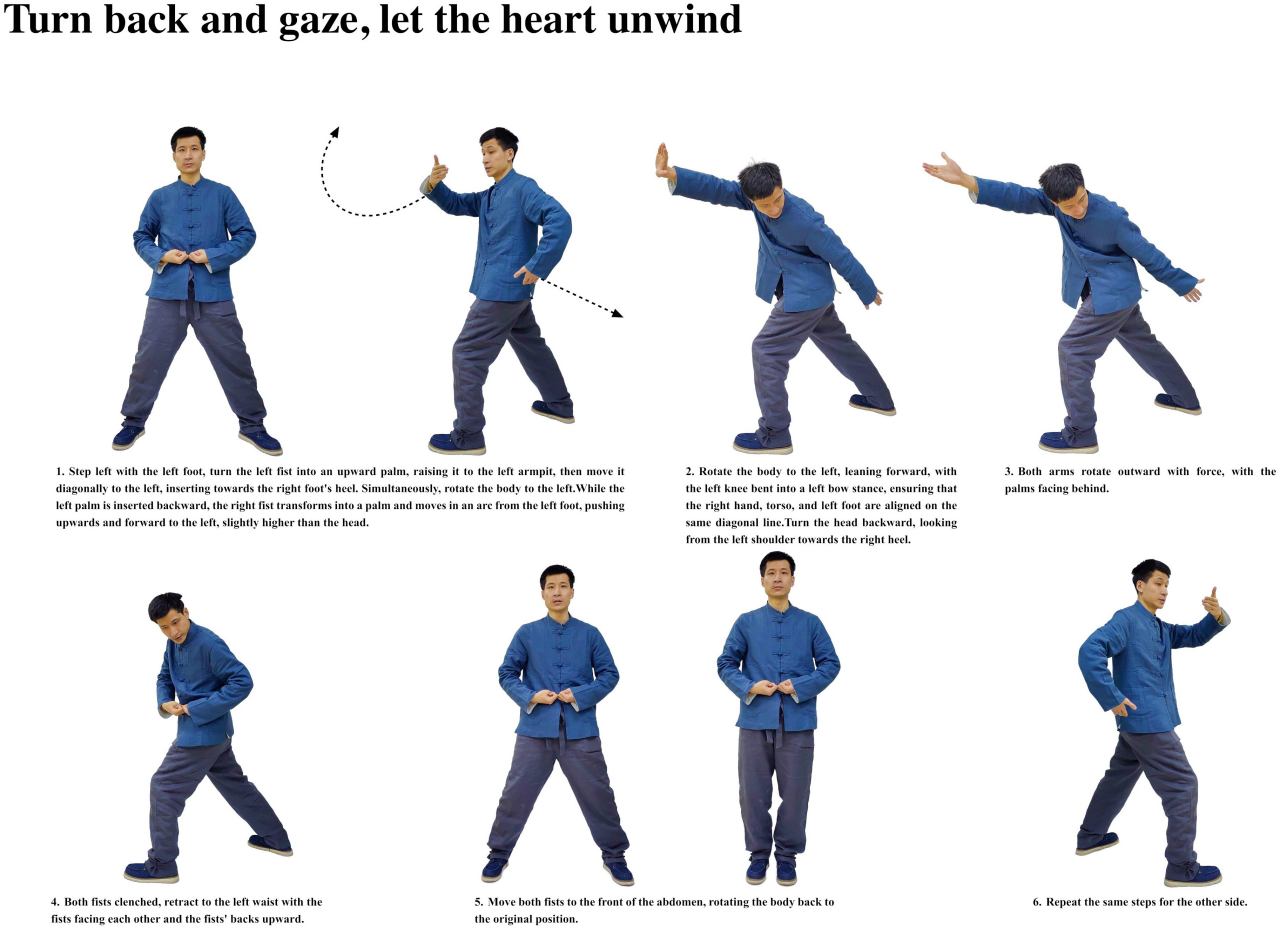
Turn back and gaze, let the heart unwind.

**FIGURE 6:**
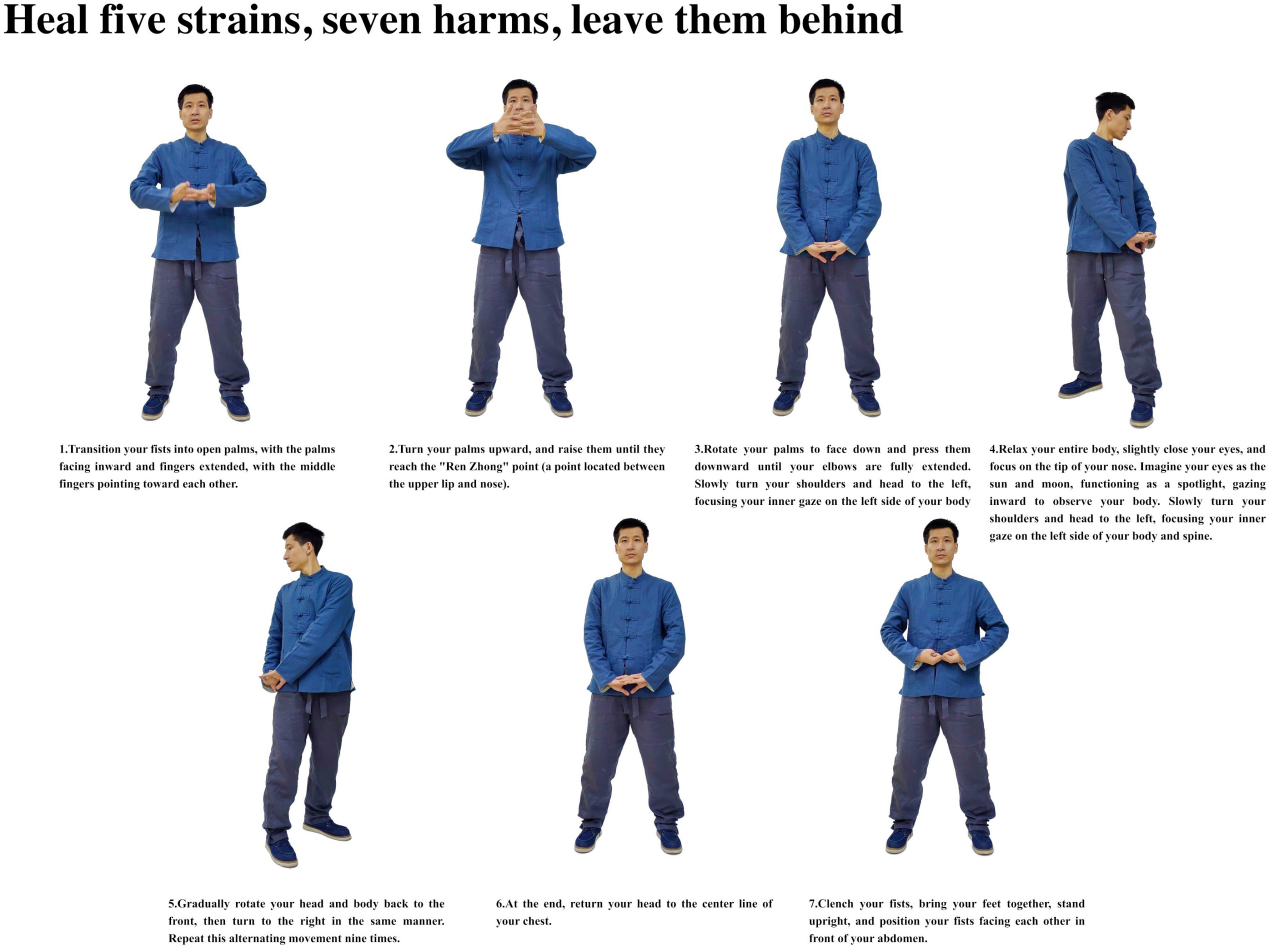
Heal five strains, seven harms, leave them behind.

**FIGURE 7:**
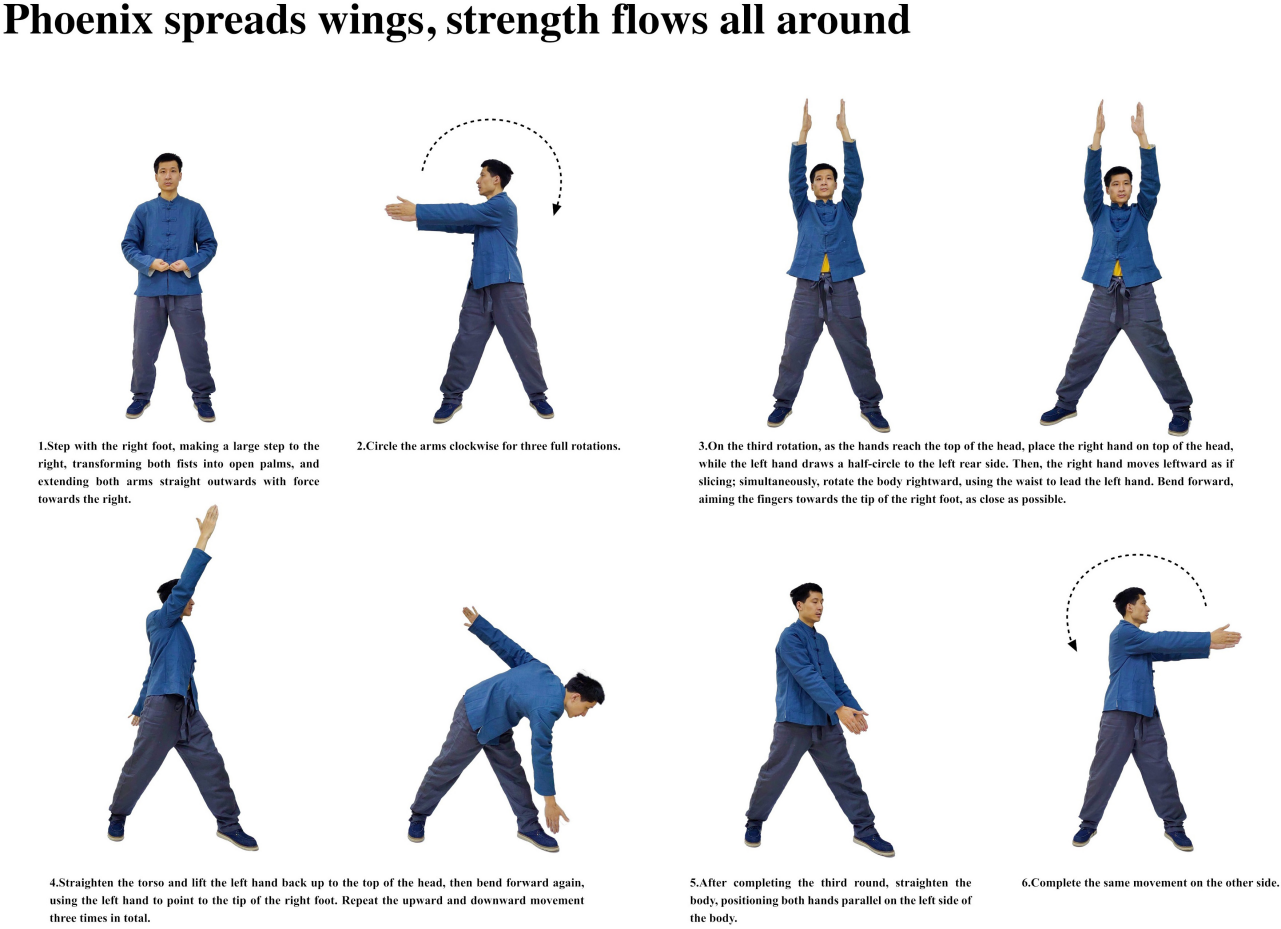
Phoenix spreads wings, strength flows all around.

**FIGURE 8:**
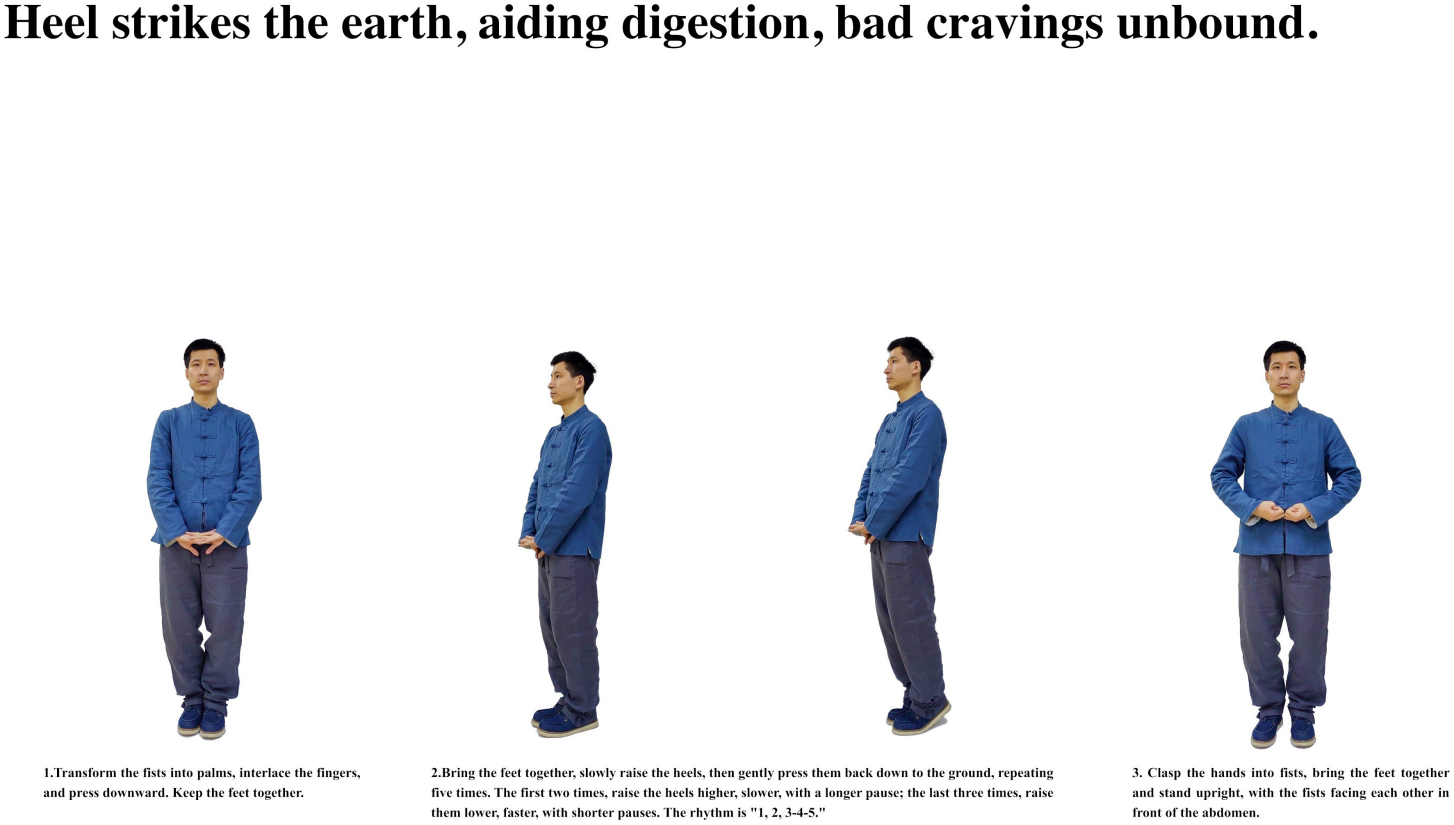
Heel strikes the earth, aiding digestion, bad cravings unbound.

Participants with insomnia completed 12 weeks of ESVE training before follow-up MRI scans, with weekly exercise duration documented in minutes. The ESVE exercise protocol followed a standardized sequence as illustrated in the practice flow diagrams (Figures 1–8), which feature demonstration photographs of the study author who provided written informed consent for publication and use of these images. ESVE instructors were not blinded to the study objectives; however, neuroimaging data acquisition and analysis personnel remained blinded to group assignments and clinical outcomes to minimize potential bias in data processing and interpretation.

The Ethics Committee of Guangdong Provincial Hospital of Traditional Chinese Medicine approved the study protocol, and all participants provided written informed consent.

### 2.2 Neuroimaging data acquisition

MRI images were acquired using a 3.0 T MR scanner (MAGNETOM Prisma, Siemens, Germany) equipped with a 64-channel head coil. T2-weighted and T2-FLAIR sequences were routinely obtained to rule out organic brain lesions. For the rs-fMRI images, the echo planar sequence was employed with the following parameters: field of view (FOV) = 244 × 244 mm, echo time (TE) = 30 ms, repetition time (TR) = 500 ms, thickness = 3.5 mm, voxel size = 3.5 × 3.5 × 3.5 mm, slices = 35, flip angle = 60°, and a total of 960 volumes were acquired, resulting in a total scan duration of 8 min (960 volumes × 0.5 s TR = 480 s).

The parameters for the 3D T1WI sequence are as follows: inversion time (TI) = 1,100 ms, TR = 2,530 ms, TE = 2.98 ms, FOV = 256 × 256 mm, thickness = 1 mm, voxel size = 1 × 1 × 1 mm, flip angle = 7°, and a total of 192 sagittal slices. All participants were instructed to close their eyes and remain conscious during the scan.

### 2.3 Data preprocessing

#### 2.3.1 Gray matter volume analysis

VBM of T1-weighted structural MRI (sMRI) images was calculated using the Computational Anatomy Toolbox, CAT12^1^ together with SPM12.^2^ VBM included segmentation of images into gray matter, white matter, and cerebrospinal fluid, normalization using DARTEL, and smoothing of GMV segments using an 6-mm full-width half-maximum (FWHM) isotropic Gaussian kernel.

#### 2.3.2 Rs-fMRI data preprocessing

The rs-fMRI data underwent preprocessing using the DPABI software^3^ package and were processed in MATLAB 2022b using the following steps: (1) removing the first 10 time points, (2) head motion correction, (3) spatial normalization to the Montreal Neurological Institute (MNI) space, (4) spatial smoothing with an isotropic Gaussian kernel with full width at half-maximum (FWHM) of 6 mm, (5) eliminating the linear trend of the time course, (6) regression of head motion effect, gray matter, white matter, and cerebrospinal fluid signals from the fMRI data, and (7) bandpass filtering (0.01–0.08 Hz). For images with a shorter echo time, performing slice timing during preprocessing was deemed unnecessary (Smith et al., 2013). Participants with head motion exceeding 3 mm or rotation exceeding 3° during scanning were excluded.

#### 2.3.3 ALFF and fALFF calculation

After data preprocessing, the time course of each voxel was transformed into the frequency domain using a fast Fourier transform, and the power spectrum was subsequently obtained. The square root was calculated at each frequency of the power spectrum, and the average square root was obtained as the ALFF value in the range of 0.01 m was Hz for each voxel, which was further divided by the global mean ALFF of each individual for group comparison. Divide the ALFF value by the total power to obtain the fALFF value for each voxel (Zang et al., 2007).

#### 2.3.4 ReHo calculation

A single ReHo map was generated by calculating the Kendalldalle Kendall mean ALFF of ea (KCC) of the time series of a given voxel and its nearest neighbor (26 voxels) in a voxel-wise way. The formula for calculating the KCC value has been clarified in previous studies. For standardization, the ReHo value of every voxel was divided by the global mean ReHo of each individual. The spatial smoothing (FWHM = 6 mm) was performed after ReHo calculation.

#### 2.3.5 DC calculation

Pearson’s correlation of time series was performed between each voxel and every other voxel in the entire brain to calculate a correlation matrix R = (rij), j = 1 … N (N is the number of voxels), i ≠ 1. The correlation coefficients with rij ≥ 0.32 (*P* < 0.05, Bonferroni-corrected over wholebrain voxels) were summed up for each voxel and then a weighted DC was obtained for each voxel. The weighted DC of each voxel was further divided by the global mean weighted DC of each individual for group comparison (Zhao et al., 2019; Zuo et al., 2012).

#### 2.3.6 Seed-based whole-brain FC analysis

Seed-based FC analysis was carried out to explore the alterations of PMWI. We selected brain regions that are commonly altered with ALFF, fALFF, and ReHo at baseline, along with the right superior temporal gyrus as a seed region. According to a previous study, the right superior temporal gyrus was a vital region in women insomnia patients (Zhou et al., 2017). These ROIs were specifically defined for seed-based FC analysis, while all other metrics (GMV, ALFF, fALFF, ReHo, DC) were calculated using whole-brain voxel-wise approaches. We made a 6 mm spherical region of interest (ROI) centered in the bilateral precentral gyrus (MNI space: –48, –4, 44/48, –4, 44), left paracentral lobule (MNI space: –6, –24, 60) and right superior temporal gyrus (MNI space: 52, –34, 12). Then, we extracted the mean time course from the seed regions, and Pearson’s correlation coefficients were calculated to correlate these time courses with whole-brain voxels. Finally, the FC maps were normalized into z score maps by Fisher Z-transformation.

## 3 Statistical analysis

Statistical analyses were performed using R software (version 4.4.2, R Foundation for Statistical Computing, Vienna, Austria). The normality of continuous variables was assessed using the Shapiro-Wilk test. Independent samples *t*-tests were conducted to compare age, education years, and BMI between the two groups. Paired *t*-tests were employed to evaluate changes in clinical measures (PSQI, ISI, GAD-7, PHQ-9, MoCA, and FSS scores) before and after treatment. Functional MRI data were processed and analyzed using DPABI software. We first calculated voxel-wise metrics reflecting local spontaneous neural activity (ALFF, fALFF, and ReHo), whole-brain functional network connectivity strength (DC), and seed-based functional connectivity (FC). All functional and structural metrics (ALFF, fALFF, ReHo, DC, and GMV) were compared both within and between groups at baseline and after treatment using independent samples *t*-tests (or Mann-Whitney U tests for non-normally distributed data), with age and education years as covariates for functional metrics, and an additional covariate of total intracranial volume (TIV) for structural metrics. Seed-based functional connectivity analysis also employed independent samples *t*-tests with age and education as covariates. To control for multiple comparisons, significant clusters were determined at the whole-brain level using Gaussian random field (GRF) correction (voxel-level *P* < 0.001, cluster-level *P* < 0.05, two-tailed tests).

Partial correlation analyses were performed to examine the relationships between clinical measures (PSQI, ISI, GAD-7, PHQ-9, MoCA, and FSS scores) and neuroimaging parameters. For functional measures (ALFF, fALFF, ReHo, and FC), age and education years were included as covariates. For structural measures (GMV), age, education years, and total intracranial volume (TIV) were included as covariates. The significance level was set at *P* < 0.05 (two-tailed). To investigate the association between brain functional alterations and treatment efficacy, insomnia patients were stratified into responder and non-responder groups based on the reduction rate (RR) of PSQI scores. Treatment response was defined as both RR ≥ 50% and an absolute reduction in total PSQI score ≥ 6 points. Patients who did not meet these criteria (RR < 50% or reduction in total PSQI score < 6 points) were classified as non-responders. Clinical characteristics and changes in neuroimaging parameters (ALFF, fALFF, ReHo, DC, FC, and GMV) were compared between the responder and non-responder groups.

## 4 Results

### 4.1 Analysis of demographics and clinical characteristics

**FIGURE 9:**
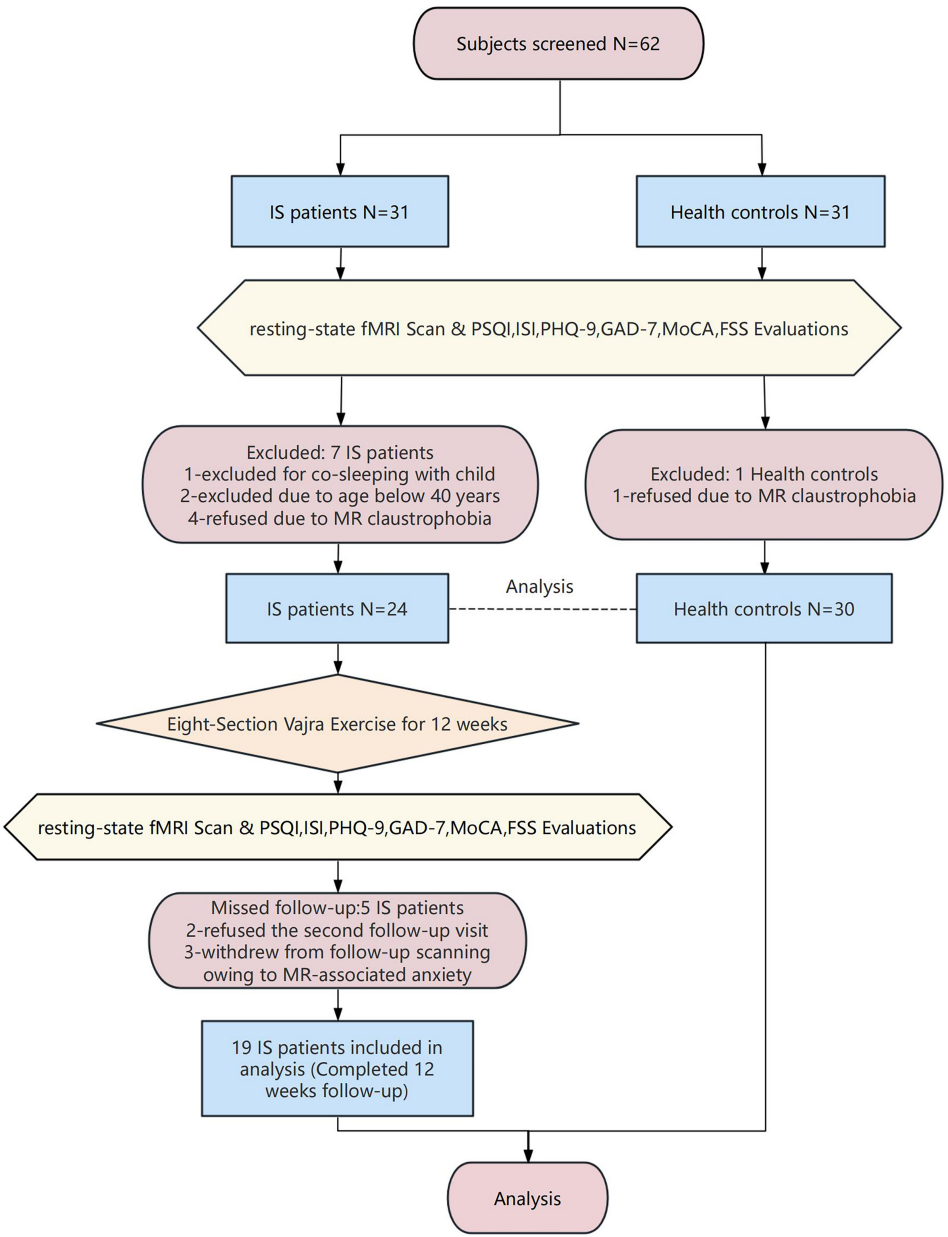
Chart flow for the study subjects.

The flowchart of study subjects is shown in Figure 9. Initially, 31 PMWI patients and 31 HCs were recruited for the study. After screening, 24 PMWI patients and 30 HCs met the inclusion criteria and were included in the statistical analyses. Sociodemographic and clinical data of the study groups are presented in Table 1, with detailed demographic data and clinical characteristics for response and non-response groups of PMWI patients provided in Supplementary Table 1. Among the 24 patients diagnosed with insomnia, 5 dropped out during the study period, leaving 19 patients for the final statistical analysis. At baseline, 12 patients (63.16%) were medication-naive. After 12 weeks of ESVE, a total of 15 patients (78.95%) were medication-free, including both the initially medication-naive patients and those who discontinued their sleep medication during the intervention. Allowed medications for PMWI patients included short-acting hypnotics used ≤ 3 times per week, with a minimum 48-h washout period before MRI scanning. Excluded medications included long-acting benzodiazepines, antidepressants with sedating effects, and any medications known to significantly alter brain connectivity patterns. Detailed information regarding specific medication use is presented in Supplementary Table 2.

**TABLE 1:** Demographic data and clinical characteristics.

Characteristics	*PMWI (n* = 24)	HCs (*n* = 30)	t/z	*P-*value
Age (years old)	58.46 ± 5.3	56.17 ± 5.58	–1.53	0.131
Education (years)	12.21 ± 2.17	10.83 ± 3.03	–1.87	0.067
Body mass index (kg/m^2^)	22.92 ± 2.86	24.45 ± 2.97	1.92	0.061
Disease duration (mo)	55.83 ± 8.44	N/A	N/A	N/A
PSQI scores	14.54 ± 2.98	3.87 ± 1.94	–15.88	< 0.001
ISI scores	17.42 ± 2.43	4.3 ± 2.15	–21.01	< 0.001
GAD-7 scores	7.38 ± 3.28	5.67 ± 2.04	–2.34	0.023
PHQ-9 scores	8.58 ± 3.24	5.4 ± 2.5	–4.08	< 0.001
MoCA scores	27.54 ± 1.44	27.23 ± 1.07	–0.9	0.372
FSS scores	44 ± 10.28	20.8 ± 7.54	–9.57	< 0.001

Unless otherwise indicated, data are means ± standard deviation; PMWI, postmenopausal women with insomnia; HCs, healthy controls; PSQI, Pittsburgh Sleep Quality Index; ISI, Insomnia Severity Index; GAD-7, Generalized Anxiety Disorder-7; PHQ-9, Patient Health Questionnaire-9; MoCA, Montreal Cognitive Assessment; FSS, Fatigue Severity Scale.

### 4.2 Rs-fMRI data comparison between PMWI and HCs at baseline

**FIGURE 10:**
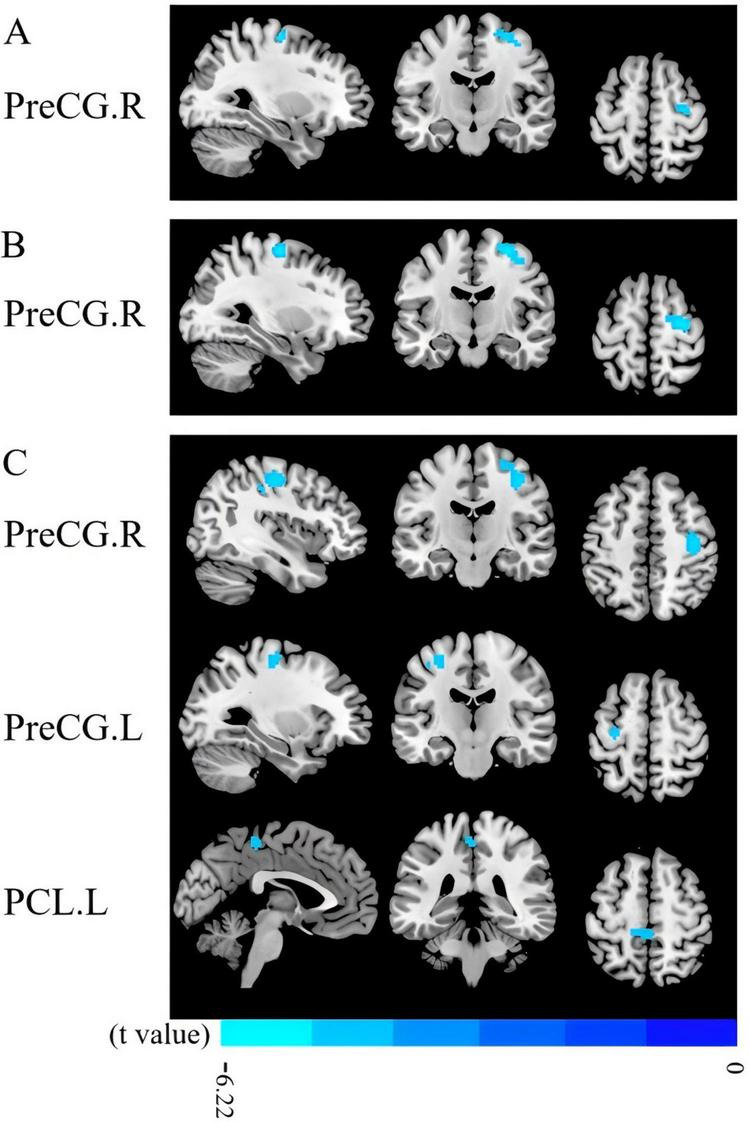
Results of ALFF, fALFF, ReHo between PMWI and HCs. **(A)** Regions with significant differences in ALFF between PMWI and HCs (GFR-corrected *P*<0.05). **(B)** Regions with significantly difference in fALFF between PMWI and HCs (GFR-corrected *P*< 0.05). **(C)** Regions with significantly difference in ReHo between PMWI and HCs (GFR-corrected *P*< 0.05). ALFF, amplitude of low-frequency fluctuations; fALFF, fractional Amplitude of low frequency fluctuations; ReHo, Regional Homogeneity; GFR, Gaussian Filter Regularization; L, left; R, right; PreCG, precentral gyrus; PCL, paracentral lobule; PMWI, Postmenopausal women with insomnia; HCs, healthy controls; PMWI<HC in blue.

Compared with HCs at baseline, PMWI patients had lower ALFF and fALFF values in the right precentral gyrus (PreCG) (*t* = –5.17/–6.22, *P* < 0.05, GRF corrected) (Figures 10A,B; Supplementary Tables 3, 4). PMWI patients exhibited lower ReHo in the bilateral PreCG (*t* = –4.49/–5.34, *P* < 0.05, GRF corrected), and the left Paracentral lobule (PCL) (*t* = –4.41, *P* < 0.05, GRF corrected) compared with HCs (Figure 10C; Supplementary Table 5). No regions showed significant differences between PMWI and HCs in terms of DC.

**FIGURE 11:**
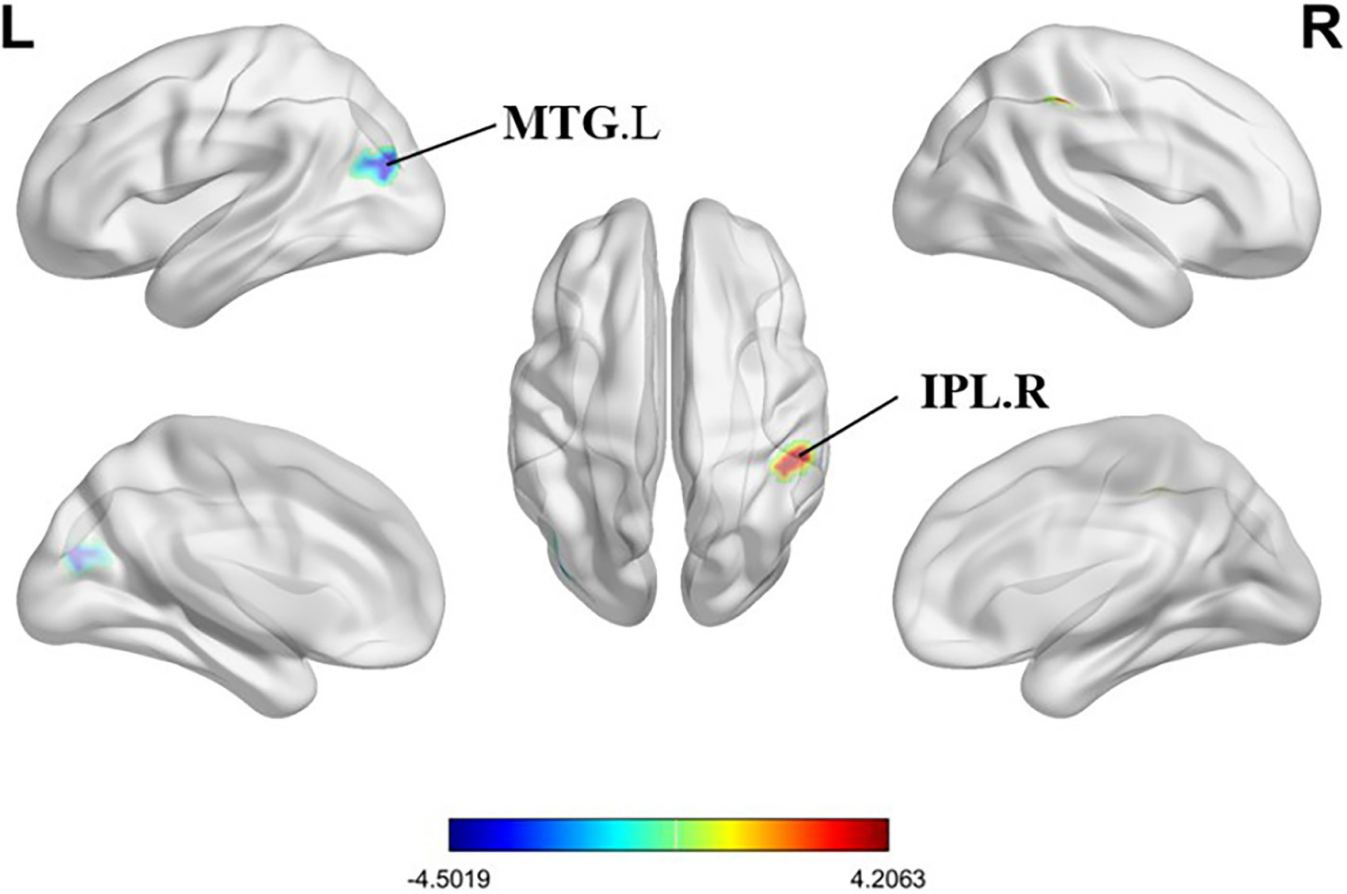
Differences in PreCG.L based functional connectivity (FC) between PMWI and HCs (GFR-corrected *P*< 0.05). The FC strengths that were increased are marked in red, while those that were decreased are marked in blue.

**FIGURE 12:**
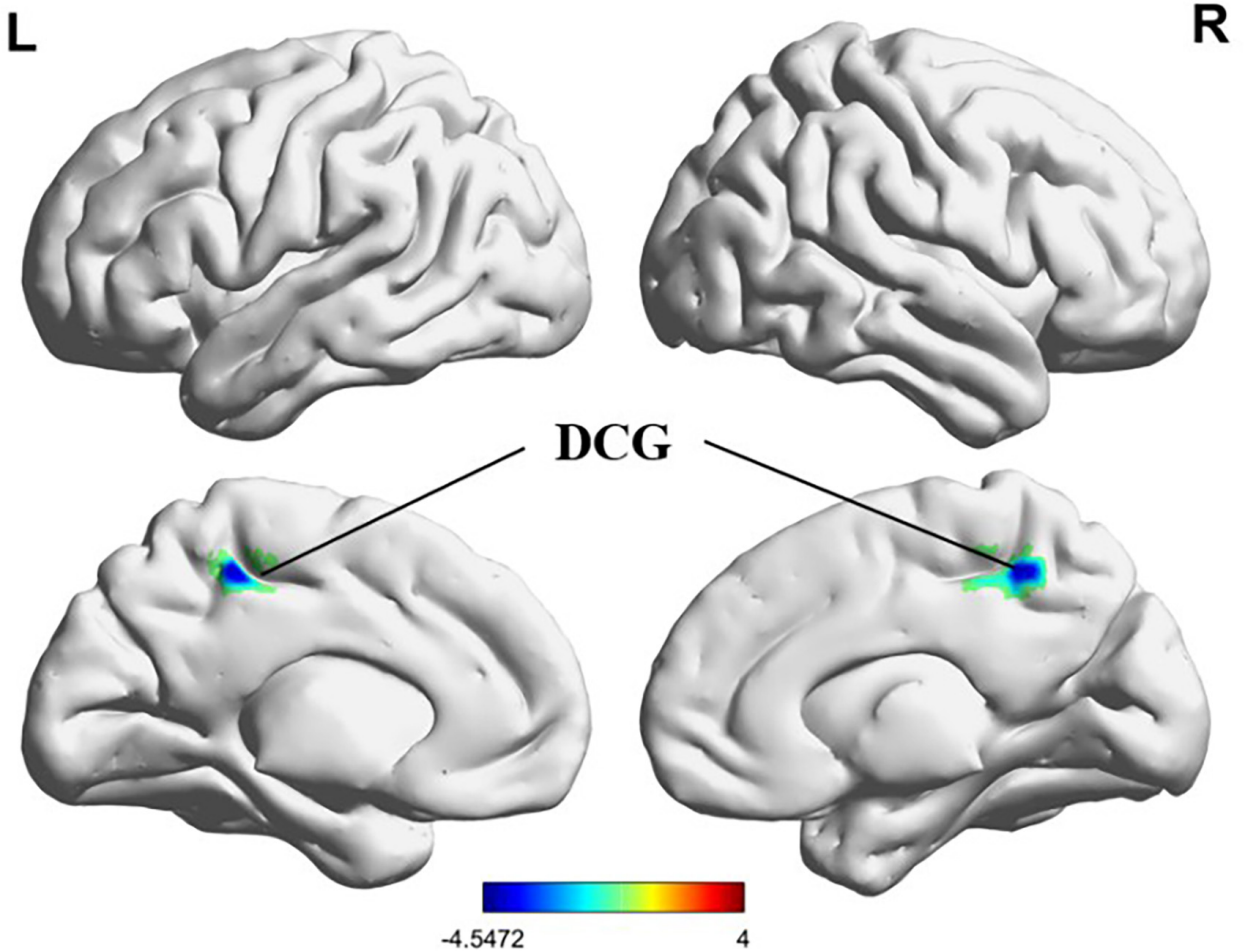
Differences in PCL.L based FC between PMWI and HCs (GFR-corrected *P*< 0.05). All the FC strengths were decreased and marked in blue.

**FIGURE 13:**
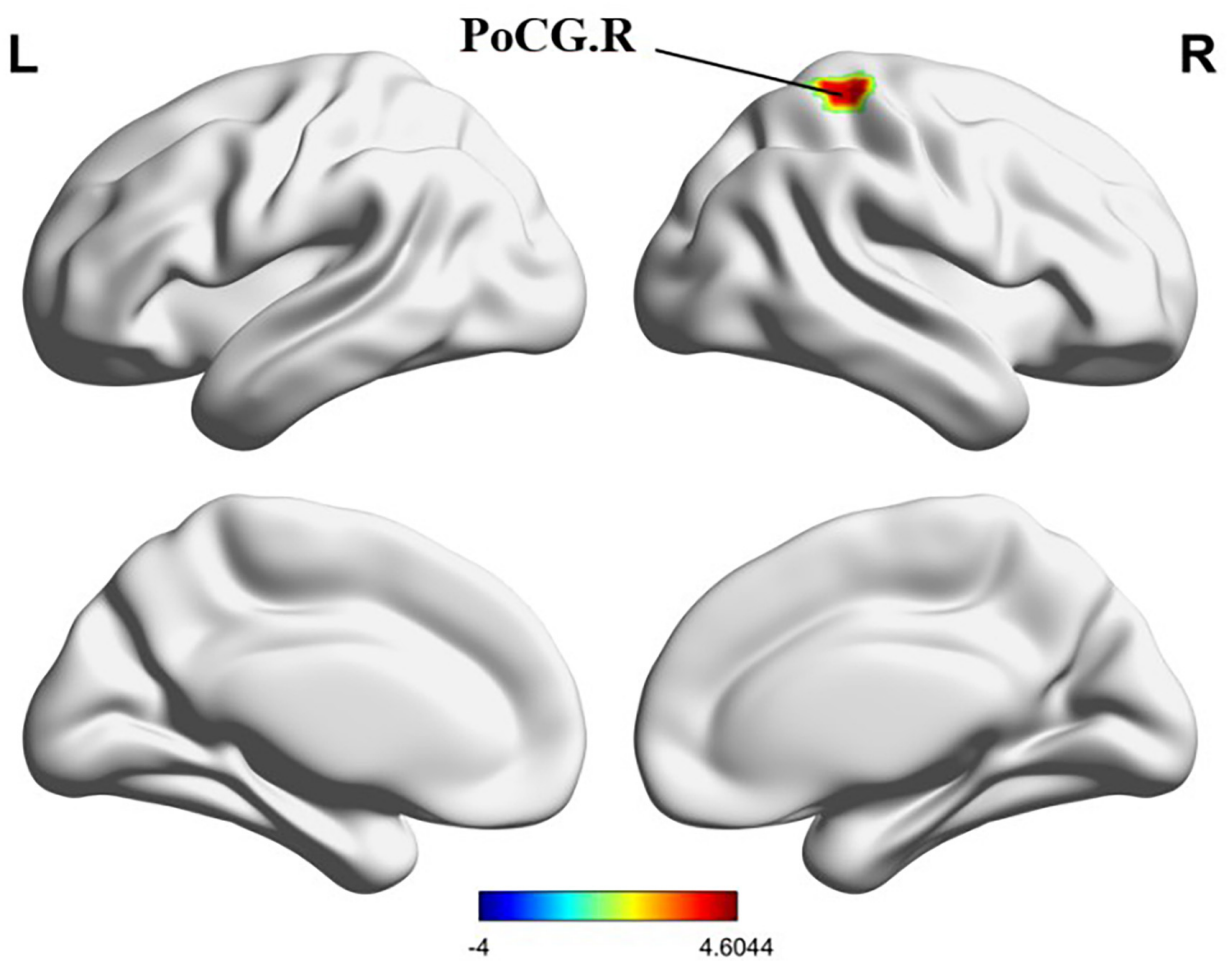
Differences in STG.R based FC between PMWI and HCs (GFR-corrected *P*< 0.05). All the FC strengths were increased and marked in red.

Compared with HCs, PMWI exhibited increased FC between the left PreCG and right inferior parietal lobule (IPL) and between the right superior temporal gyrus (STG) and the right postcentral gyrus (PoCG). Additionally, reduced FC was observed between the left PreCG and the left middle temporal gyrus (MTG), and between the left PCL and the bilateral dorsal cingulate gyrus (DCG). No FC changes were observed in the right PreCG (GRF-corrected, *P* < 0.05) (Figures 11, 12; Supplementary Table 6). Moreover, FC enhancement was observed between the left PreCG and right IPL and between the right superior temporal gyrus (STG) and the right PoCG in the PMWI group (GRF-corrected, *P* < 0.05) (Figures 11, 13; Supplementary Table 6).

### 4.3 Rs-fMRI data comparison before and after treatment in PMWI

**FIGURE 14:**
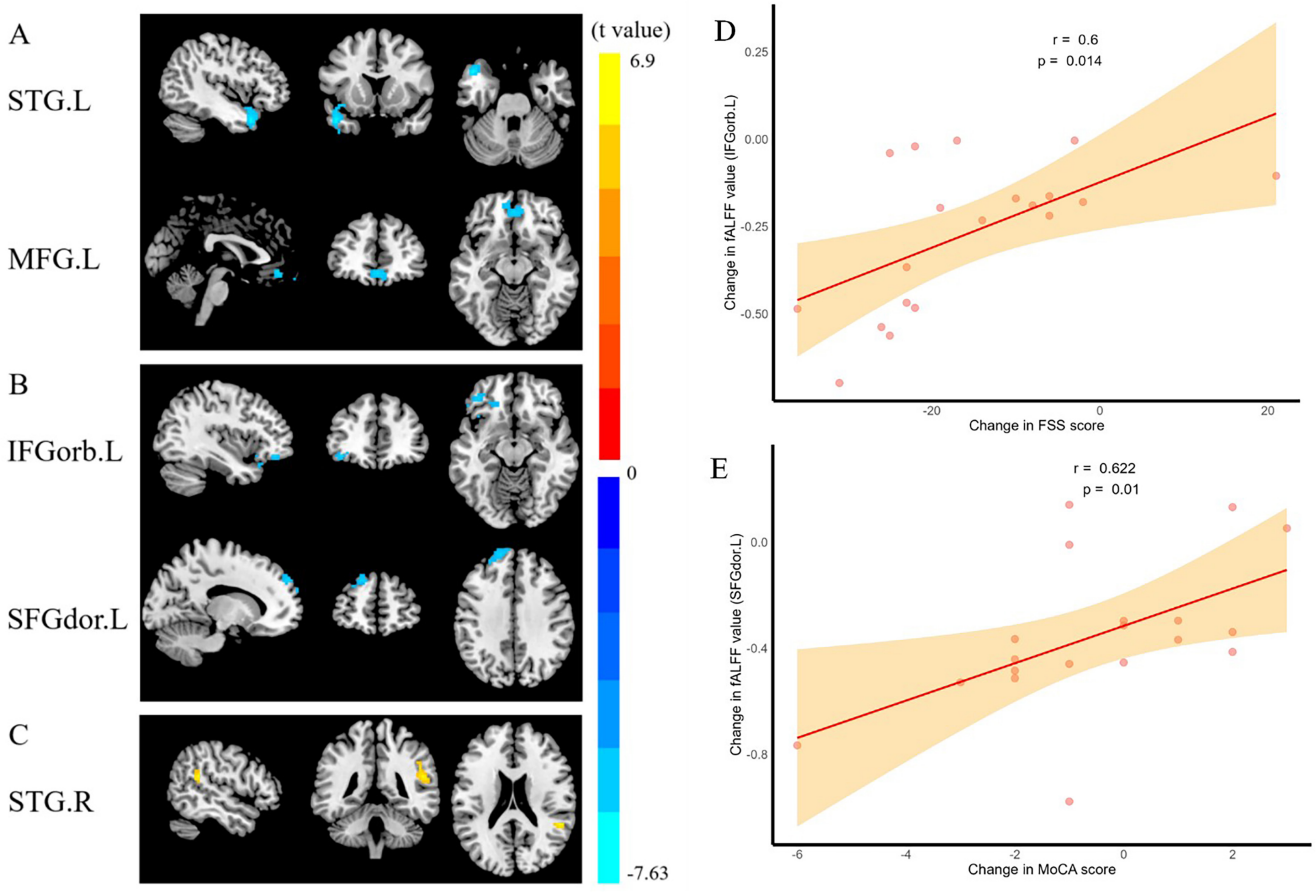
Comparison and correlation results of ALFF, fALFF, ReHo before and after treatment of PMWI. **(A)** Differences in ALFF before and after treatment (GFR-corrected *P*< 0.05). **(B)** Differences in fALFF before and after treatment (GFR-corrected *P*< 0.05). **(C)** Differences in ReHo before and after treatment (GFR-corrected *P*< 0.05). **(D)** After 12 weeks of treatment, altered fALFF of IFGorb.L significantly correlated with increase in FSS score. **(E)** After treatment, altered fALFF of IFGorb.L significantly correlated with increase in MoCA score. Note: ALFF, amplitude of low-frequency fluctuations; fALFF, fractional Amplitude of low frequency fluctuations; ReHo, Regional Homogeneity; GFR, Gaussian Filter Regularization; L, left; R, right; STG, Superior Temporal Gyrus; MFG, Middle Frontal Gyrus; Inferior Frontal Gyrus, Orbital part; SFGdor, superior frontal gyrus, dorsolateral; PMWI, Postmenopausal women with insomnia; HCs, healthy controls; Post->pretreatment in STG.R; Post-<pretreatment in STG.L, MFG.L, IFGorb.L and SFGdor.L; FSS, Fatigue Severity Scale; MoCA, Montreal Cognitive Assessment.

After treatment, PMWI patients showed decreased ALFF in the left STG (*t* = –7.64, *P* < 0.05, GRF corrected) and left medial frontal gyrus (MFG) (*t* = –5.46, *P* < 0.05, GRF corrected) (Figure 14A; Supplementary Table 3), and decreased fALFF in the left orbital part of Inferior frontal gyrus (IFGorb) (*t* = –5.14, *P* < 0.05, GRF corrected), the left dorsolateral region of superior frontal gyrus (SFGdor) (*t* = –5.43, *P* < 0.05, GRF corrected) (Figure 14B; Supplementary Table 4). Conversely, ReHo was in the right STG (*t* = 6.90, *P* < 0.05, GRF corrected) (Figure 14C; Supplementary Table 5). In contrast, no differences in DC were observed.

**FIGURE 15:**
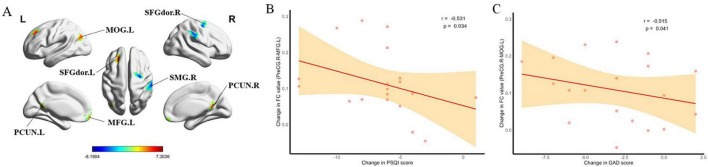
Comparison and correlation results of FC before and after treatment of PMWI. **(A)** Differences in PreCG.R based FC before and after treatment (GFR-corrected *P*< 0.05). **(B)** After 12 weeks of treatment, altered FC of PreCG.R-MFG.L significantly correlated with decrease in PSQI score. **(C)** After treatment, altered FC of PreCG.R-MCG.L significantly correlated with decrease in GAD score.

**FIGURE 16:**
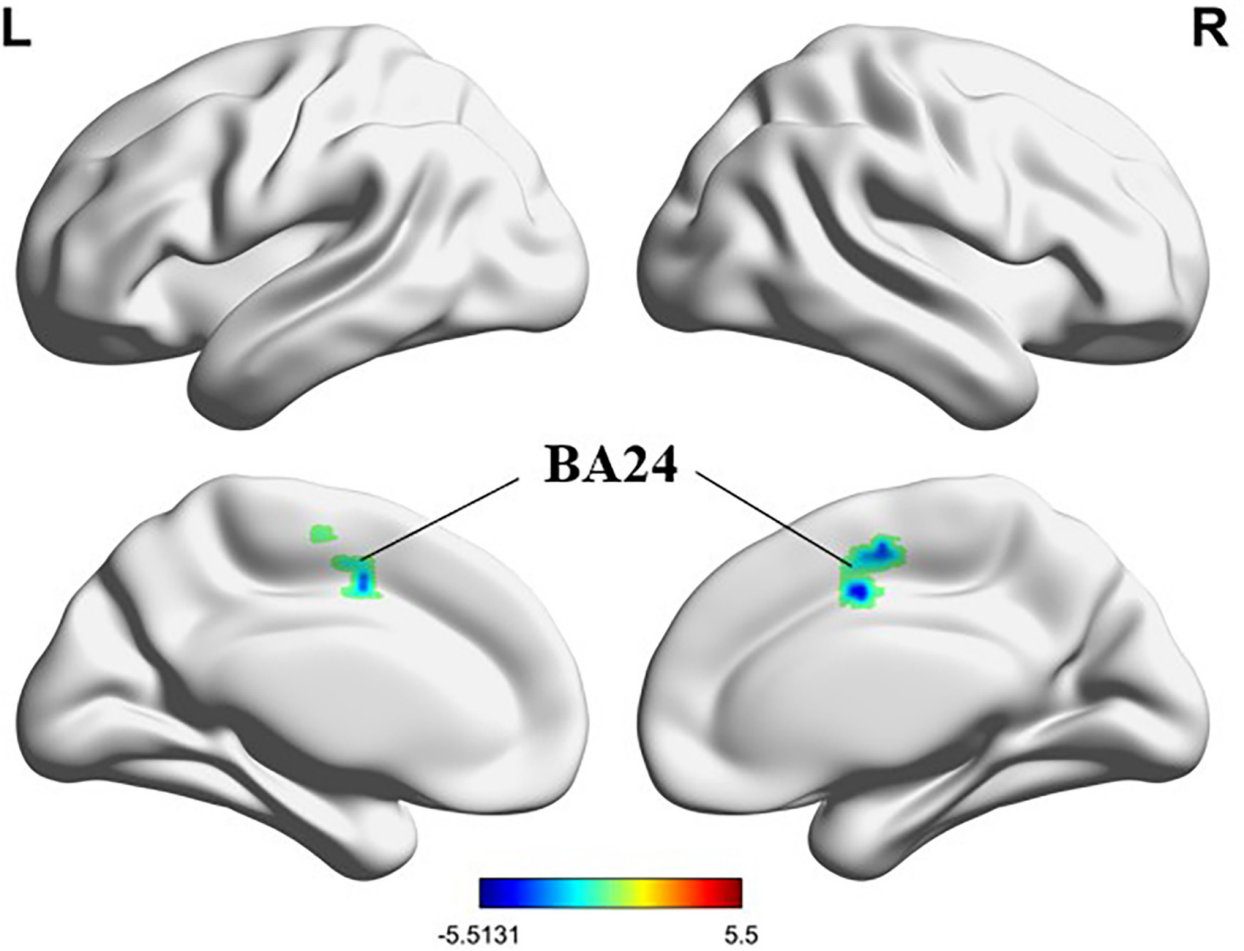
Differences in STG.R based FC before and after treatment (GFR-corrected *P* < 0.05). All the FC strengths were decreased and marked in blue.

After treatment, the PMWI group showed no FC changes in the left PreCG and the left PCL, whereas reduced FC was observed between the right PreCG and the right Supramarginal gyrus (SMG) as well as the right superior frontal gyrus dorsal part (SFGdor.R), and between the right STG and the Brodmann area 24 (GRF-corrected, *P* < 0.05) (Figures 15, 16; Supplementary Table 6). Moreover, FC enhancement was observed between the PreCG.R and the brain regions including the left medial frontal gyrus (MFG.L), Precuneus (PCUN), the left of Middle occipital gyrus (MOG.L) and SFGdor.L (GRF-corrected, *P* < 0.05) (Figure 15; Supplementary Table 6).

### 4.4 Gray matter volume comparison between patients with PMWI and HCs at baseline

Compared with HCs, PMWI showed lower GMV in the left SFGdor (*t* = –4.11, *P* < 0.05, GRF corrected) and increased GMV in the left MTG (*t* = 3.71, *P* < 0.05, GRF corrected) (Supplementary Figure 1).

### 4.5 Gray matter volume comparison before and after treatment in PMWI

After treatment, Regions with increased GMV in PMWI were found in the left Inferior temporal gyrus (ITG) (*t* = 5.75, *P* < 0.05, GRF corrected) and the left Cuneus (*t* = 5.58, *P* < 0.05, GRF corrected) (Supplementary Table 7).

### 4.6 Partial correlation analysis between clinical measures and imaging parameters

The partial correlation analysis revealed notable associations between clinical measures and imaging parameters. Changes in fALFF within IFGorb.L after treatment were positively correlated with FSS score changes (*r* = 0.600, *P* = 0.014) (Figure 14D), while significant positive correlations were observed in SFGdor.L with MoCA score changes (*r* = 0.622, *P* = 0.010) (Figure 14E). In contrast, no significant correlations were identified for ALFF, ReHo, and GMV values before and after treatment (Supplementary Table 8).

Furthermore, FC value changes in PreCG.R-MFG.L demonstrated significant negative correlations with PSQI score changes (*r* = –0.531, *P* = 0.034) (Figure 15B; Supplementary Table 8). Similarly, FC changes from PreCG.R-MCG.L were negatively correlated with GAD score changes (*r* = –0.515, *P* = 0.041) (Figure 15C; Supplementary Table 8). Additionally, correlation analyses of ESVE against various measures revealed significant positive correlations, particularly with PSQI RR (*r* = 0.508, *P* = 0.044) and FC value of PreCG.R-MOG.L (*r* = 0.594, *P* = 0.015) (Supplementary Table 9).

### 4.7 Differences between the response and non-response group

Of the 19 patients with PMWI, 9 (47.37%) were classified as Response and 10 (52.63%) as Non-response to ESVE intervention. The average weekly practice duration of ESVE was significantly different between the two groups (*P* < 0.05). Post-intervention analysis revealed that the response demonstrated significantly improved PSQI scores, particularly in domains of subjective sleep quality, sleep latency, sleep duration, and sleep efficiency, as well as ISI scores compared to the Non-response. However, no significant differences were observed in other PSQI components, GAD-7 scores, PHQ-9 scores, FSS scores, or MoCA scores between the groups (*P* > 0.05) (Supplementary Table 1). No significant changes were found in ALFF, fALFF, ReHo, DC, FC, and GMV in both groups before and after treatment (*P* > 0.05).

## 5 Discussion

This study reveals a novel neuroplastic mechanism underlying insomnia improvement in postmenopausal women through Eight-Section Vajra Exercise (ESVE), with the precentral gyrus (PreCG) emerging as a critical neural hub. The key finding demonstrates that enhanced PreCG-middle occipital gyrus (MOG) functional connectivity correlates with sleep quality improvement, suggesting an integrative mechanism bridging motor regulation and visual processing networks for sleep-related memory consolidation. Notably, the PreCG’s dual functionality in motor inhibition during non-rapid eye movement (NREM) sleep and motor memory consolidation was found to be particularly vulnerable to estrogen fluctuations, offering a neurobiological explanation for postmenopausal insomnia susceptibility. The intervention-induced gray matter volume increases in visual processing regions (inferior temporal gyrus and cuneus) coupled with PreCG-centered network reorganization highlight exercise-mediated neuroplastic adaptations. Clinically significant correlations emerged between exercise dosage (253 vs. 189 min/week in responders vs. non-responders) and sleep improvement, positioning ESVE as a viable non-pharmacological intervention. This work innovatively bridges traditional exercise therapy with modern neuroimaging biomarkers, establishing PreCG-centered sensorimotor-visual network modulation as both a mechanistic pathway and therapeutic target for hormonally-mediated insomnia.

Our findings underscore the PreCG’s pivotal role in both motor control and sleep-related memory consolidation. The convergence of alterations in ALFF, fALFF, and ReHo metrics within this region highlights its significance in understanding the neurobiological underpinnings of insomnia, especially in vulnerable populations such as postmenopausal women, who often experience sleep disturbances linked to hormonal fluctuations and neurophysiological changes (Tal et al., 2015; Hestiantoro et al., 2019; Haufe and Leeners, 2023). This spatial convergence not only reinforces the robustness of our findings but also emphasizes the PreCG’s critical role in the pathophysiology of insomnia.

The PreCG, traditionally associated with motor control, exhibits a dual functionality in sleep, wherein it suppresses voluntary movements while simultaneously facilitating the consolidation of motor memory (Wang et al., 2022; Vahdat et al., 2017). Research indicates that during non-rapid eye movement (NREM) sleep, the brain engages in processes that enhance motor memory consolidation, which is crucial for learning new skills (Brawn et al., 2010; Nitsche et al., 2010; Mantua et al., 2016). The activity of sleep spindles, characterized by bursts of oscillatory brain activity, has been shown to correlate with improved performance in motor tasks, suggesting that these spindles play a vital role in the offline consolidation of motor skills (Fogel et al., 2017; Mander et al., 2017).

In the context of postmenopausal women, the functions of the PreCG appear particularly susceptible to fluctuations in estrogen levels. This hormonal variability may contribute to the observed dysregulation within the motor system and associated sleep disturbances (Xu et al., 2023). Studies have shown that estrogen influences the neural circuitry involved in both motor control and sleep regulation, suggesting that hormonal changes could exacerbate the challenges faced by this demographic in maintaining effective sleep patterns and motor function (Chou et al., 2021). The interplay between hormonal fluctuations and neural activity in the PreCG may thus provide a comprehensive understanding of the mechanisms driving insomnia and its related motor dysfunctions.

Our study also revealed significant structural and functional connectivity alterations in postmenopausal insomnia patients. Specifically, we observed a decrease in gray matter volume (GMV) in the left superior frontal gyrus (SFGdor.L) and an increase in GMV in the left middle frontal gyrus (MFG) at baseline. Additionally, the functional connectivity between the PreCG.L and left middle temporal gyrus (MTG.L) was reduced, while connectivity between the PreCG.L and right inferior parietal lobule (IPL.R) increased. These findings suggest a complex reorganization of neural networks in response to insomnia (Weber et al., 2013; Chen et al., 2017). Moreover, the paracentral lobule (PCL) plays a critical role in regulating muscle tone and suppressing voluntary movements, thereby promoting deep relaxation and facilitating the body’s functional recovery.

Our study found that the ReHo of the PCL was significantly reduced in postmenopausal insomnia patients compared to healthy controls, indicating potential dysfunction within this core component of the sensorimotor network (SMN) (Otte et al., 2015). The observed decrease in functional connectivity between the PCL and dorsal cingulate gyrus (DCG) reflects a dysfunction within the default mode network (DMN), which is associated with self-referential processing and resting-state cognition. This impairment may lead to disrupted sleep patterns and cognitive functions (Sugijantoro et al., 2022). The significance of these findings is further underscored by the complex interplay between the Central Executive Network (CEN) and DMN, which represents a critical adaptation for maintaining cognitive stability under sleep disruption. The reduced functional connectivity between the PCL and DCG suggests a breakdown in the neural mechanisms that typically support the integration of cognitive and emotional processes during sleep, essential for effective memory consolidation and emotional regulation (Moradi Farsani et al., 2021). This dysfunction may exacerbate the challenges faced by postmenopausal women, who are already at heightened risk for insomnia and related cognitive impairments due to hormonal fluctuations (Bojar et al., 2022; Kling et al., 2017). The hormonal variability experienced by this demographic could contribute to the observed dysregulation within the motor system and associated sleep disturbances, highlighting the need for targeted interventions that address both the psychological and hormonal aspects of insomnia in postmenopausal women (Masoudi et al., 2020).

The present study investigates the structural and functional alterations in postmenopausal female insomnia patients following an intervention of ESVE. Notably, the results indicate significant changes in both functional connectivity and gray matter volume (GMV) in specific brain regions, which may correlate with improvements in sleep quality. One of the most compelling findings is the enhanced functional connectivity between the right precentral gyrus (PreCG.R) and the left middle occipital gyrus (MOG.L). This increase in connectivity is particularly relevant as it may suggest a mechanism through which the ESVE contributes to improved sleep. The PreCG is involved in motor control, while the MOG plays a role in visual processing. Enhanced connectivity between these areas could indicate a more integrated neural network that supports better sleep regulation, potentially alleviating insomnia symptoms in postmenopausal women. This aligns with previous research that highlights the importance of functional connectivity in sleep disorders, suggesting that improved connectivity may facilitate better sleep maintenance and initiation (Emamian et al., 2021, 2019).

Additionally, the observed increases in GMV in the left inferior temporal gyrus (ITG) and cuneus are noteworthy. The ITG is associated with visual processing and memory, while the cuneus is involved in visual perception and attention. An increase in GMV in these regions may reflect neuroplastic changes resulting from the ESVE intervention, which could enhance cognitive functions related to sleep. The significance of these alterations cannot be understated, as they may indicate a compensatory mechanism in response to the cognitive demands placed on individuals suffering from insomnia. Enhanced GMV in these areas could also correlate with improved emotional regulation and stress management, which are critical factors in the context of insomnia (Masoudi et al., 2021).

The ESVE, as a form of traditional Chinese exercise therapy, may exert its effects through multiple pathways. The exercise promotes relaxation, reduces stress, and enhances overall wellbeing, which are essential for improving sleep quality in postmenopausal women. The simplicity and accessibility of ESVE make it particularly suitable for this demographic, as it requires less dynamic balance and cognitive load compared to other forms of exercise like Tai Chi. The physiological benefits of regular physical activity, such as improved circulation and reduced muscle tension, may further contribute to the observed changes in brain structure and function (Lampio et al., 2014; Yang et al., 2018).

Notably, the changes in functional connectivity between the PreCG.R and MFG.L regions were negatively correlated with the Pittsburgh Sleep Quality Index (PSQI) improvement. This suggests that enhanced connectivity in these areas may be associated with better sleep quality outcomes. Previous research has indicated that the PreCG is involved in motor control and cognitive processes, which could explain its role in sleep regulation and quality (Creasy et al., 2019). The negative correlation observed implies that as functional connectivity improves, sleep quality also enhances, reinforcing the importance of targeted interventions like the ESVE in managing insomnia symptoms.

The relationship between exercise volume and treatment efficacy was also evident in our results. The response group demonstrated a significantly higher weekly exercise duration (253.14 ± 69.47 min) compared to the non-response group (189.39 ± 53.22 min), with a *p*-value of 0.037. This finding aligns with existing literature that emphasizes the role of physical activity in improving sleep quality and reducing insomnia severity (Hartescu et al., 2015; Luo et al., 2023). Regular physical exercise has been shown to enhance sleep through various mechanisms, including the modulation of circadian rhythms and the reduction of stress levels, which are critical factors in insomnia management (Rozales et al., 2024; Fan et al., 2021). Moreover, the psychological benefits of exercise, such as improved mood and reduced anxiety, may further contribute to sleep quality improvements (Karandikar-Agashe and Agrawal, 2020; Passos et al., 2014).

Additionally, we observed distinct characteristics between the response and non-response groups. While demographic factors such as age, education, and body mass index did not significantly differ, the response group exhibited lower insomnia severity scores (ISI) and higher FSS scores post-intervention. This suggests that the response group not only experienced improved sleep quality but also enhanced functional status, which is crucial for overall wellbeing in postmenopausal women (Masoudi et al., 2021). The findings indicate that the response group may have better coping mechanisms or resilience against insomnia, potentially influenced by their higher engagement in physical activity.

This study has several limitations. First, the relatively small sample size (*n* = 19 completers) may limit generalizability. Second, the 12-week intervention period precludes assessment of long-term neuroplastic changes. Third, the absence of an active control group (e.g., other exercise modalities) prevents direct comparison of ESVE-specific effects. Fourth, methodological limitations include lack of polysomnographic confirmation and reliance solely on voxel-based morphometry without diffusion-weighted imaging. Fifth, hormonal levels were not systematically measured, leaving estrogen-neuroplasticity interactions unquantified. Future studies should incorporate: (1) Larger multicenter cohorts with longitudinal follow-up, (2) Comprehensive sleep assessment including polysomnography, (3) Multimodal imaging including diffusion tensor imaging, (4) Comparative effectiveness trials against established interventions, (5) Hormonal assay integration, and (6) Task-based fMRI paradigms.

## 6 Conclusion

This multimodal neuroimaging study demonstrates that 12 weeks of ESVE induces functional reorganization of PreCG-centered networks and structural plasticity in visual processing regions in postmenopausal insomnia patients. The dose-dependent relationship between exercise adherence and sleep improvement, coupled with PreCG-MOG connectivity enhancement, suggests this traditional intervention may mitigate hormonally-mediated neural vulnerability through sensorimotor-visual integration. These findings position ESVE as a viable non-pharmacological strategy for postmenopausal insomnia, with neuroplastic changes in motor control regions serving as potential biomarkers for treatment response. The results bridge traditional exercise therapy with modern neuroimaging, providing empirical support for movement-based interventions in managing endocrine-related sleep disorders.

## Data Availability

The original contributions presented in this study are included in this article/Supplementary material, further inquiries can be directed to the corresponding authors.

## Footnotes

1
http://dbm.neuro.uni-jena.de/cat/

2
http://www.fil.ion.ucl.ac.uk/spm/

3
http://www.restfmri.net/forum/DPABI
