# Supplementary material for: Structural and functional alterations in postmenopausal women with insomnia: an MRI study of Eight-Section Vajra Exercise intervention effects
Source: Front Neurosci. 2026 Jan 30;19:1622756. doi: 10.3389/fnins.2025.1622756 (PMC12901484; doi:10.3389/fnins.2025.1622756)
Supplement: Supplementary file 1 [file Data_Sheet_1.docx]

**
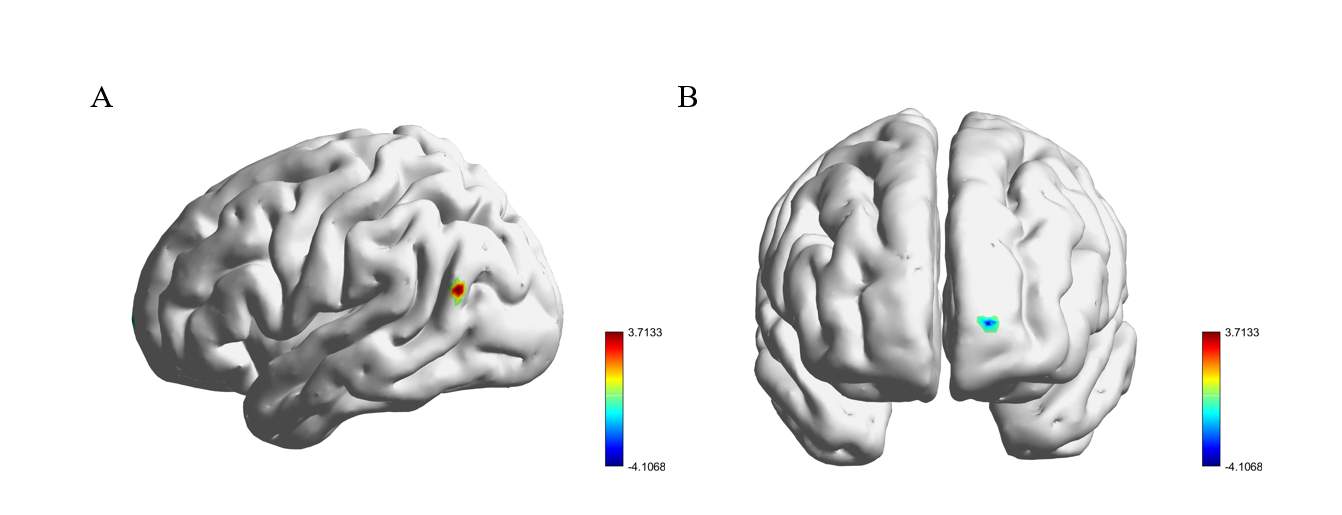
**

**Supplementary figure 1.** Results of gray matter volume between PMWI and HCs. (A) The MTG.L showed increased GMV compared to HCs. (B) The SFG.L showed decreased GMV compared to HCs.
