# Supplementary material for: Structural and functional alterations in postmenopausal women with insomnia: an MRI study of Eight-Section Vajra Exercise intervention effects
Source: Front Neurosci. 2026 Jan 30;19:1622756. doi: 10.3389/fnins.2025.1622756 (PMC12901484; doi:10.3389/fnins.2025.1622756)
Supplement: Supplementary file 2 [file Data_Sheet_2.zip › Table/Supplementary Table 1. Demographic data and clinical characteristics of response group and non-response group.docx]

| **Supplementary Table 1** Demographic data and clinical characteristics of response group and non-response group | | | | | | |
| --- | --- | --- | --- | --- | --- | --- |
| **Characteristics** | **Response group (n=9)** | | **Non-response group (n=10)** | | **t/z** | **P Value** |
| Age (years old) | 59.67 ± 5.92 | | 57.3 ± 4.74 | | -0.97 | 0.347 |
| Education (years) | 11.78 ± 2.17 | | 12.7 ± 2.16 | | 0.93 | 0.367 |
| Body Mass Index (kg/㎡) | 24.16 ± 3.96 | | 22.66 ± 1.81 | | -1.08 | 0.295 |
| Disease duration (mo) | 58.28 ± 12.08 | | 55.05 ± 5.22 | | -0.77 | 0.452 |
| Exercise(min/week) | 253.14 ± 69.47 | | 189.39 ± 53.22 | | -2.26 | 0.037 |
|  | **Baseline** | **12 Week** | **Baseline** | **12 Week** |  |  |
| PSQI scores | 13.78 ± 2.05 | 5 ± 1.94 | 15.4 ± 3.69 | 11.1 ± 2.13^**^ |  |  |
| Subjective Sleep Quality | 2.33 ± 0.5 | 0.78 ± 0.44 | 2.7 ± 0.67 | 1.3 ± 0.48^**^ |  |  |
| Sleep Latency | 2.67 ± 0.5 | 0.67 ± 0.5 | 2.6 ± 0.84 | 2.2 ± 0.79^**^ |  |  |
| Sleep Duration | 1.56 ± 0.53 | 0.89 ± 0.6 | 2.3 ± 1.06 | 2.2 ± 0.79^**^ |  |  |
| Sleep Efficiency | 1.89 ± 0.93 | 0.44 ± 0.53 | 2.6 ± 0.7 | 2.5 ± 0.97^**^ |  |  |
| Sleep Disturbances | 2.22 ± 0.44 | 1 ± 0.5 | 1.6 ± 0.52 | 1.2 ± 0.42 |  |  |
| Use of Sleeping Medication | 0.78 ± 1.09 | 0.33 ± 1 | 1.2 ± 1.55 | 0.9 ± 1.45 |  |  |
| Daytime Dysfunction | 2.33 ± 1 | 0.89 ± 0.33 | 2.4 ± 0.7 | 0.8 ± 0.42 |  |  |
| ISI scores | 17.44 ± 2.07 | 5.22 ± 3.56 | 17.9 ± 2.64 | 11.1 ± 3.84^*^ |  |  |
| GAD-7 scores | 6.67 ± 3.28 | 3.56 ± 3.36 | 8.2 ± 3.26 | 5.2 ± 2.74 |  |  |
| PHQ-9 scores | 8 ± 3.16 | 4.67 ± 2.4 | 9.3 ± 3.09 | 5.3 ± 2.21 |  |  |
| MoCA scores | 27.33 ± 1 | 27.22 ± 1.56 | 28.2 ± 1.32 | 27.3 ± 2.11 |  |  |
| FSS scores | 43.67 ± 12.02 | 25.78 ± 11.17 | 43.2 ± 10.4 | 29.6 ± 10.62 |  |  |

Note: Unless otherwise indicated, data are means ± standard deviation; *P < 0.05; **P < 0.001; PSQI, Pittsburgh Sleep Quality Index; ISI, Insomnia Severity Index; GAD-7, Generalized Anxiety Disorder-7; PHQ-9, Patient Health Questionnaire-9; MoCA, Montreal Cognitive Assessment; FSS, Fatigue Severity Scale.
