# Supplementary material for: Structural and functional alterations in postmenopausal women with insomnia: an MRI study of Eight-Section Vajra Exercise intervention effects
Source: Front Neurosci. 2026 Jan 30;19:1622756. doi: 10.3389/fnins.2025.1622756 (PMC12901484; doi:10.3389/fnins.2025.1622756)
Supplement: Supplementary file 2 [file Data_Sheet_2.zip › Table/Supplementary Table 2. Medication use in 19 Postmenopausal women with insomnia patients at baseline and at 12 weeks.docx]

| **Supplementary Table 2** Medication use in 19 postmenopausal women with insomnia patients at baseline and at 12 weeks | | | | | |
| --- | --- | --- | --- | --- | --- |
|  | Baseline | |  | 12 week | |
|  | N | Ratio(%) |  | N | Ratio(%) |
| No medication | 12 | 63.16 |  | 15 | 78.95 |
| Benzodiazepines | 5 | 26.32 |  | 3 | 15.79 |
| Non - Benzodiazepines | 1 | 5.26 |  | 0 | 0.00 |
| SSRI | 1 | 5.26 |  | 1 | 5.26 |

Note: Data are presented as numbers and percentages. SSRI, Selective Serotonin Reuptake Inhibitors.
