# Supplementary material for: Structural and functional alterations in postmenopausal women with insomnia: an MRI study of Eight-Section Vajra Exercise intervention effects
Source: Front Neurosci. 2026 Jan 30;19:1622756. doi: 10.3389/fnins.2025.1622756 (PMC12901484; doi:10.3389/fnins.2025.1622756)
Supplement: Supplementary file 2 [file Data_Sheet_2.zip › Table/Supplementary Table 4. Regions with significant difference in fALFF.docx]

**Supplementary Table 4** Regions with significant difference in fALFF

|  | Regions | side | Cluster size | Peak coorainates(MIN) | | | t |
| --- | --- | --- | --- | --- | --- | --- | --- |
|  |  |  |  | x | y | z |  |
| Baseline |  |  |  |  | | |  |
| PMWI<HC | Precentral gyrus | R | 112 | 30 | -12 | 63 | -6.2161 |
|  |  |  |  |  | | |  |
| 12 weeks |  |  |  |  | | |  |
| Post-<pre-treatment | Inferior frontal gyrus, orbital part | L | 81 | -39 | 42 | -12 | -5.1422 |
|  | Superior frontal gyrus, dorsolateral | L, | 79 | -15 | 54 | 36 | -5.4275 |

Note: GRF-corrected (P < 0.001 voxel-level, P < 0.05 cluster-level). Peak coordinates refer to the point with the highest t value in the cluster, not the specific region; x, y, z coordinates of peak locations in the Montreal Neurological Institute space (MNI); fALFF, fractional Amplitude of Low-Frequency Fluctuations; PMWI, postmenopausal women with insomnia; HC, healthy control; L, Left; R, Right.
