# Supplementary material for: Structural and functional alterations in postmenopausal women with insomnia: an MRI study of Eight-Section Vajra Exercise intervention effects
Source: Front Neurosci. 2026 Jan 30;19:1622756. doi: 10.3389/fnins.2025.1622756 (PMC12901484; doi:10.3389/fnins.2025.1622756)
Supplement: Supplementary file 2 [file Data_Sheet_2.zip › Table/Supplementary Table 5. Regions with significant difference in ReHo.docx]

**Supplementary Table 5** Regions with significant difference in ReHo

|  | Regions | side | Cluster size | Peak coorainates(MIN) | | | t |
| --- | --- | --- | --- | --- | --- | --- | --- |
|  |  |  |  | x | y | z |  |
| Baseline |  |  |  |  | | |  |
| PMWI<HC | Precentral Gyrus | R | 119 | 39 | -15 | 51 | -5.3406 |
|  | Precentral Gyrus | L | 45 | -30 | -18 | 60 | -4.4947 |
|  | Paracentral lobule | L | 40 | -3 | -36 | 57 | -4.4119 |
| 12 weeks |  |  |  |  | | |  |
| Post->pre-treatment | Superior Temporal Gyrus | R | 33 | 51 | -42 | 21 | 6.9 |
|  |  |  |  |  |  |  |  |

Note: GRF-corrected (P < 0.001 voxel-level, P < 0.05 cluster-level). Peak coordinates refer to the point with the highest t value in the cluster, not the specific region; x, y, z coordinates of peak locations in the Montreal Neurological Institute space (MNI); ReHo, Regional Homogeneity; PMWI, postmenopausal women with insomnia; HC, healthy control; L, Left; R, Right.
