# Supplementary material for: Structural and functional alterations in postmenopausal women with insomnia: an MRI study of Eight-Section Vajra Exercise intervention effects
Source: Front Neurosci. 2026 Jan 30;19:1622756. doi: 10.3389/fnins.2025.1622756 (PMC12901484; doi:10.3389/fnins.2025.1622756)
Supplement: Supplementary file 2 [file Data_Sheet_2.zip › Table/Supplementary Table 6. Differences in FC between PMWI and healthy controls.docx]

**Supplementary Table 6** Differences in FC between PMWI and healthy controls

|  | Seed  area | Seed coordinates | | | Cluster size | Connected location | Peak coorainates(MIN) | | | t |
| --- | --- | --- | --- | --- | --- | --- | --- | --- | --- | --- |
|  |  |  |  |  |  |  | x | y | z |  |
| Baseline |  |  | | |  |  |  |  |  |  |
|  | PreCG.L | -48 | -4 | 44 | 71 | MTG.L | -36 | -69 | 18 | -4.5019 |
|  |  |  |  |  | 57 | IPL.R | 51 | -33 | 48 | 4.2063 |
|  | PCL.L | -6 | -24 | 60 | 52 | DCG | 0 | -36 | 51 | -4.5472 |
|  | STG.R | 52 | -34 | 12 | 66 | PoCG.R | 36 | -39 | 63 | 4.6044 |
| 12 weeks |  |  |  |  |  |  |  |  |  |  |
|  | PreCG.R | 48 | -4 | 44 | 116 | MFG.L | -3 | 66 | -15 | 7.3036 |
|  |  |  |  |  | 128 | PCUN | -9 | -54 | 12 | 6.7577 |
|  |  |  |  |  | 95 | MOG.L | -36 | -84 | 36 | 6.1279 |
|  |  |  |  |  | 100 | SFGdor.L | -24 | 33 | 42 | 6.4968 |
|  |  |  |  |  | 83 | SMG.R | 57 | -30 | 42 | -5.5134 |
|  |  |  |  |  | 65 | SFGdor.R | 30 | -6 | 57 | -8.1904 |
|  | STG.R | 52 | -34 | 12 | 76 | BA24 | 6 | -6 | 51 | -5.5131 |

Note: GRF-corrected (P < 0.001 voxel-level, P < 0.05 cluster-level). Peak coordinates refer to the point with the highest t value in the cluster, not the specific region; x, y, z coordinates of peak locations in the Montreal Neurological Institute space (MNI); FC, Functional Connectivity; PMWI, postmenopausal women with insomnia; HC, healthy control; L, Left; R, Right.
