# Supplementary material for: Structural and functional alterations in postmenopausal women with insomnia: an MRI study of Eight-Section Vajra Exercise intervention effects
Source: Front Neurosci. 2026 Jan 30;19:1622756. doi: 10.3389/fnins.2025.1622756 (PMC12901484; doi:10.3389/fnins.2025.1622756)
Supplement: Supplementary file 2 [file Data_Sheet_2.zip › Table/Supplementary Table 7. Regions with significant difference in GMV.docx]

**Supplementary Table 7** Regions with significant difference in GMV

|  | Regions | side | Cluster size | Peak coorainates(MIN) | | | t |
| --- | --- | --- | --- | --- | --- | --- | --- |
|  |  |  |  | x | y | z |  |
| Baseline |  |  |  |  | | |  |
| PMWI<HC | Superior frontal gyrus, dorsolateral | L | 15 | -15 | 70.5 | 3 | -4.1068 |
|  | Middle temporal gyrus | L | 38 | -54 | -66 | 21 | 3.7133 |
| 12 weeks |  |  |  |  | | |  |
| Post->pre-treatment | Inferior temporal gyrus | L | 27 | -43.5 | -28.5 | -22.5 | 5.7468 |
|  | Cuneus | L | 12 | -15 | -78 | 37.5 | 5.5849 |

Note: GRF-corrected (P < 0.001 voxel-level, P < 0.05 cluster-level). Peak coordinates refer to the point with the highest t value in the cluster, not the specific region; x, y, z coordinates of peak locations in the Montreal Neurological Institute space (MNI); GMV, Gray Matter Volume; PMWI, postmenopausal women with insomnia; HC, healthy control; L, Left; R, Right.
