# Supplementary material for: Structural and functional alterations in postmenopausal women with insomnia: an MRI study of Eight-Section Vajra Exercise intervention effects
Source: Front Neurosci. 2026 Jan 30;19:1622756. doi: 10.3389/fnins.2025.1622756 (PMC12901484; doi:10.3389/fnins.2025.1622756)
Supplement: Supplementary file 2 [file Data_Sheet_2.zip › Table/Supplementary Table 8. Partial Correlation analysis between clinical data and ALFF,fALFF,ReHo,GMV and FC.docx]

| **Supplementary Table 8** Partial Correlation analysis between clinical data and ALFF,fALFF,ReHo,GMV and FC | | | | | | | |
| --- | --- | --- | --- | --- | --- | --- | --- |
| Variables | ALFF | | fALFF | | Reho | GMV | |
|  | MFG.L(r/P) | STG.L(r/P) | IFGorb.L(r/P) | SFGdor.L(r/P) | STG.R(r/P) | ITG.L(r/P) | Cuneus_L(r/P) |
| PSQI | 0.424/0.102 | -0.013/0.963 | 0.228/0.395 | 0.068/0.802 | 0.306/0.25 | -0.272/0.328 | -0.509/0.052 |
| SSQ | 0.111/0.682 | 0.137/0.612 | 0.018/0.947 | 0.381/0.145 | 0.412/0.113 | -0.107/0.705 | 0.113/0.689 |
| SL | 0.091/0.737 | 0.093/0.732 | -0.021/0.938 | -0.172/0.525 | -0.154/0.57 | -0.222/0.426 | **-0.623/0.013** |
| SD | 0.485/0.057 | 0.115/0.672 | 0.174/0.519 | -0.063/0.817 | -0.048/0.861 | -0.247/0.374 | -0.091/0.746 |
| SE | 0.192/0.476 | -0.222/0.408 | -0.06/0.826 | -0.068/0.802 | 0.148/0.584 | -0.143/0.61 | **-0.594/0.02** |
| SDist | 0.412/0.112 | 0.174/0.518 | 0.425/0.101 | 0.424/0.101 | 0.343/0.193 | **-0.716/0.003** | **-0.653/0.008** |
| USM | 0.059/0.828 | -0.38/0.147 | 0.116/0.67 | -0.403/0.122 | 0.034/0.901 | 0.413/0.126 | 0.175/0.532 |
| DD | 0.231/0.39 | 0.122/0.654 | 0.297/0.263 | 0.276/0.302 | 0.426/0.1 | 0.033/0.908 | 0.123/0.663 |
| ISI | 0.259/0.333 | -0.047/0.862 | 0.273/0.306 | 0.137/0.613 | 0.376/0.151 | -0.233/0.404 | -0.469/0.077 |
| PHQ | -0.133/0.623 | -0.002/0.996 | 0.017/0.95 | 0.115/0.671 | -0.22/0.413 | -0.037/0.896 | 0.327/0.235 |
| GAD | -0.311/0.241 | 0.096/0.723 | 0.051/0.852 | 0.099/0.716 | 0.066/0.808 | -0.159/0.572 | 0.054/0.848 |
| MoCA | 0.402/0.122 | 0.398/0.127 | 0.46/0.073 | **0.622/0.01** | 0.215/0.425 | -0.403/0.136 | -0.175/0.532 |
| FSS | 0.332/0.209 | -0.325/0.219 | **0.6/0.014** | 0.394/0.131 | 0.278/0.297 | -0.113/0.687 | -0.136/0.629 |

| Variables | | FC | | | | | | |
| --- | --- | --- | --- | --- | --- | --- | --- | --- |
|  |  | PreCG.R-MFG.L(r/P) | PreCG.R-PCUN(r/P) | PreCG.R-MOG.L(r/P) | PreCG.R-SFGdor.L(r/P) | PreCG.R-SMG.R(r/P) | PreCG.R-SFGdor.R(r/P) | STG.R-BA24(r/P) |
| PSQI | | **-0.531/0.034** | -0.019/0.943 | 0.01/0.972 | 0.108/0.689 | 0.105/0.7 | 0.135/0.618 | 0.088/0.746 |
| SSQ | | 0.167/0.535 | 0.191/0.478 | 0.198/0.462 | 0.07/0.797 | -0.065/0.812 | -0.235/0.38 | 0.101/0.709 |
| SL | | -0.46/0.073 | 0.056/0.836 | -0.08/0.768 | 0.113/0.678 | 0.25/0.351 | 0.216/0.423 | -0.219/0.416 |
| SD | | -0.348/0.187 | -0.406/0.119 | 0.045/0.869 | -0.009/0.974 | 0.091/0.736 | 0.263/0.325 | 0.296/0.265 |
| SE | | -0.256/0.34 | 0.279/0.295 | 0.111/0.682 | 0.124/0.648 | 0.195/0.469 | 0.086/0.751 | -0.044/0.87 |
| SDist | | **-0.602/0.014** | -0.15/0.578 | -0.244/0.363 | 0.084/0.757 | 0.247/0.357 | 0.303/0.254 | -0.203/0.451 |
| USM | | -0.259/0.334 | -0.238/0.374 | -0.271/0.311 | -0.214/0.425 | 0.074/0.784 | 0.181/0.502 | 0.004/0.989 |
| DD | | -0.135/0.618 | 0.049/0.856 | 0.236/0.379 | 0.193/0.474 | **-0.503/0.047** | -0.336/0.203 | 0.459/0.074 |
| ISI | | -0.473/0.064 | 0.183/0.497 | -0.255/0.341 | -0.106/0.696 | 0.293/0.27 | 0.27/0.313 | -0.143/0.597 |
| PHQ | | 0.184/0.495 | -0.254/0.343 | -0.278/0.297 | -0.493/0.053 | 0.106/0.696 | 0.268/0.315 | 0.146/0.59 |
| GAD | | 0.091/0.737 | -0.144/0.594 | **-0.515/0.041** | 0.021/0.939 | 0.233/0.385 | -0.072/0.79 | -0.367/0.162 |
| MoCA | | -0.087/0.748 | -0.023/0.932 | -0.112/0.678 | 0.087/0.749 | 0.114/0.675 | 0.162/0.55 | 0.038/0.888 |
| FSS | -0.229/0.393 | | -0.441/0.087 | 0.052/0.849 | 0.245/0.36 | -0.062/0.82 | 0.129/0.635 | -0.102/0.708 |

Note: Partial correlations were performed controlling for age and education years (functional measures) or age, education years, and total intracranial volume (structural measures). Data are presented as correlation coefficient/P-value. Bold values indicate P < 0.05. PSQI, Pittsburgh Sleep Quality Index; SSQ, subjective sleep quality; SL, sleep latency; SDu, sleep duration; SE, sleep efficiency; SD, sleep disturbances; SM, use of sleep medications; DD, daytime dysfunction; ISI, Insomnia Severity Index; GAD-7, Generalized Anxiety Disorder-7; PHQ-9, Patient Health Questionnaire-9; MoCA, Montreal Cognitive Assessment; FSS, Fatigue Severity Scale; ALFF, Amplitude of Low-Frequency Fluctuations; fALFF, fractional Amplitude of Low-Frequency Fluctuations; ReHo, Regional Homogeneity; GMV, Gray Matter Volume; FC, Functional Connectivity; MFG, Medial Frontal Gyrus; STG, Superior Temporal Gyrus; IFGorb, orbital part of Inferior Frontal Gyrus; SFGdor, dorsolateral region of Superior Frontal Gyrus; ITG, Inferior Temporal Gyrus; L, left; R, right.
