# Supplementary material for: Structural and functional alterations in postmenopausal women with insomnia: an MRI study of Eight-Section Vajra Exercise intervention effects
Source: Front Neurosci. 2026 Jan 30;19:1622756. doi: 10.3389/fnins.2025.1622756 (PMC12901484; doi:10.3389/fnins.2025.1622756)
Supplement: Supplementary file 2 [file Data_Sheet_2.zip › Table/Supplementary Table 9. Relationship between Exercise and PSQI RR, ALFF, fALFF, ReHo, GMV and FC.docx]

| Supplementary Table 9 Relationship between Exercise and PSQI RR, ALFF, fALFF, ReHo, GMV and FC | | | |
| --- | --- | --- | --- |
| Variables | | Exercise | |
|  |  | *r* | P value |
| PSQI RR |  | 0.508 | **0.044** |
| ALFF | MFG.L | -0.201 | 0.454 |
|  | STG.L | -0.024 | 0.929 |
| fALFF | IFGorb.L | -0.342 | 0.195 |
|  | SFGdor.L | 0.056 | 0.838 |
| ReHo | STG.R | -0.279 | 0.295 |
| FC | PreCG.R-MFG.L | 0.468 | 0.067 |
|  | PreCG.R-PCUN | 0.418 | 0.107 |
|  | PreCG.R-MOG.L | 0.594 | **0.015** |
|  | PreCG.R-SFGdor.L | -0.025 | 0.928 |
|  | PreCG.R-SMG.R | -0.274 | 0.305 |
|  | PreCG.R-SFGdor.R | -0.168 | 0.535 |
|  | STG.R-BA24 | 0.108 | 0.69 |
| GMV | ITG.L | 0.263 | 0.344 |
|  | Cuneus-L | 0.392 | 0.149 |

Note: Pearson correlations were performed between weekly exercise duration (minutes) and outcome measures. Data are presented as correlation coefficient (r) and P-value. Bold values indicate P < 0.05. PSQI RR, Pittsburgh Sleep Quality Index Reduction Rate; ALFF, Amplitude of Low-Frequency Fluctuations; fALFF, fractional Amplitude of Low-Frequency Fluctuations; ReHo, Regional Homogeneity; GMV, Gray Matter Volume; FC, Functional Connectivity; MFG, Medial Frontal Gyrus; STG, Superior Temporal Gyrus; IFGorb, orbital part of Inferior Frontal Gyrus; SFGdor, dorsolateral region of Superior Frontal Gyrus; ITG, Inferior Temporal Gyrus; PCUN, Precuneus; MOG, Middle Occipital Gyrus; SMG, Supramarginal Gyrus; BA24, Brodmann Area 24; L, left; R, right.
